# The national and subnational prevalence and burden of age–related macular degeneration in China

**DOI:** 10.7189/jogh.07.020703

**Published:** 2017-12

**Authors:** Peige Song, Yuhang Du, Kit Yee Chan, Evropi Theodoratou, Igor Rudan, on behalf of the Global Health Epidemiology Research Group (GHERG)

**Affiliations:** Centre for Global Health Research, Usher Institute of Population Health Sciences and Informatics, University of Edinburgh, Edinburgh, Scotland, United Kingdom

## Abstract

**Background:**

Age–related macular degeneration (AMD) is the third most common cause of blindness, and the fourth leading cause of visual impairment worldwide, but little is known about the burden of this disease in the most populous country–China. This study provides the first comprehensive estimates of the prevalence and burden of AMD in China from 1990 to 2015, with projections till 2050.

**Methods:**

In this study, a systematic review and meta–analysis was conducted to estimate the prevalence of AMD in China. China National Knowledge Infrastructure (CNKI), Wanfang, Chinese Biomedicine Literature Database (CBM–SinoMed), PubMed, Embase and Medline were searched before September 2016. Multilevel mixed–effect meta–regression was performed to define the prevalence rates of AMD and its subtypes. UN population data were used to estimate and project the number of people affected from 1990 to 2050. Based on different demographic and geographic features, the national burden of AMD in 2000 and 2010 was distributed to different regions in China.

**Results:**

Our search returned 2016 citations, of which 25 met the inclusion criteria. The prevalence of any AMD ranged from 2.44% (95% CI = 1.85–3.22) in people aged 45–49 years to 18.98% (95% CI = 15.05–23.66) in people aged 85–89 years. Prevalence of early AMD ranged from 1.79% (95% CI = 1.05–3.02) to 10.05% (95% CI = 6.17–15.97), and, in the case of late AMD, from 0.38% (95% CI = 0.16–0.97) to 3.88% (95% CI = 1.68–9.13). In late AMD, the prevalence of geographic atrophy (GA) was 0.15% (95% CI = 0.05–0.47) in people aged 45–49 years and 1.09% (95% CI = 0.35–3.36) in those aged 85–89 years, and the prevalence of neovascular AMD (NVAMD) ranged between 0.24% (95% CI = 0.11–0.50) and 2.79% (95% CI = 1.33–5.77). The number of people with any AMD was 12.01 million (95% CI = 9.29–15.46) in 1990 and 26.65 million (95% CI = 20.62–34.27) in 2015. Within the same period, the number of people with early AMD increased from 9.44 million (95% CI = 7.74–11.15) to 20.91 million (95% CI = 17.16–24.68), and those with late AMD rose from 2.58 million (95% CI = 1.56–4.30) to 5.74 million (95% CI = 3.46–9.59). In late AMD, the number of people living with GA ranged from 0.87 million (95% CI = 0.40–1.83) in 1990 to 1.93 million (95% CI = 0.89–4.08) in 2015, and NVAMD from 1.71 million (95% CI = 1.16–2.47) to 3.81 million (95% CI = 2.57–5.51). The projected number of people with any AMD in 2020 is 31.23 million (95% CI = 24.18–40.14), increasing to 55.19 million (95% CI = 43.04–70.30) in 2050. Between different regions, the South Central owed the most AMD cases (5.50 million in 2000 and 7.52 million in 2010), whereas the North–West China the least (0.66 million in 2000 and 0.95 million in 2010).

**Conclusions:**

The estimates in this study suggest a substantial burden of AMD in China, with the ageing process in Chinese society, this burden will be increasing in the foreseen future. Primary and secondary prevention and treatment and effective government response are urgently needed. Improved epidemiological studies are also required to better develop eye–care strategies and health services.

Age–related macular degeneration (AMD), a degenerative disease of the macula, is a leading cause of severe and irreversible loss of vision globally, and most notably in developed countries [1–3]. In 2010, it was estimated that AMD was the third most common cause of blindness, and the fourth leading cause of visual impairment worldwide [4]. Although AMD is not a life threatening disorder, up to one–third of the affected individuals will experience various degrees of disability and depression during the course of the disease, even when only one eye is affected [5,6]. Moreover, AMD is notably associated with falls and other injuries, resulting in increased economic and social burden for the individual, caregiver and community to bear [7–10]. Ageing is consistently documented as the most important risk factor for AMD [1,3,10]. In addition, other factors, such as cigarette smoking, female gender, ethnicity, and genetic predisposition may also play a role [3,11,12]. The combined effect of continuous exposure to different risk factors and different demographic ageing speed resulted in the global epidemic of AMD showing substantial variation across different ethnic groups and geographic regions [3,13–15].

The clinical course of AMD can be broadly divided into two stages: early and late (advanced) [1,16,17]. Early AMD is characterised by soft drusen and/or pigmentary changes, but many early cases do not progress to the advanced form [16,17]. Late AMD includes two types: geographic atrophy (GA) and neovascular (exudative) AMD (NVAMD). Compared with early AMD, late AMD is far less frequent but most damaging to the sight [18]. According to the latest global estimate of AMD prevalence, both early and late AMD were most frequent in populations of European ancestry (11.20% and 0.50%). Early AMD is least common in Asians (6.81%) while late AMD is least common in populations of African ancestry (0.28%) [3]. With Asia having the largest share of the world’s population, and understanding that AMD is an age–driven disorder, it was estimated that Asia had the greatest number of people with AMD in 2014 (59 million). Furthermore, this number is expected to increase at the fastest pace in Asia in comparison to other regions – to 113 million by 2040. China, the most populous country in the world, is experiencing the most rapid ageing trend among all developing countries. It has been estimated that more than one–third of Chinese people living in China will be aged 60 years and over by 2050 [19]. It is, therefore, important to have an up–to–date summary of the magnitude and distribution of AMD in the general population to inform stakeholders and guide eye–related health policy–making and health services allocation in China.

In the last two decades, an increasing number of epidemiological studies of AMD have been conducted in China. The estimates were, however, contingent upon the characteristics of individual studies: the age structure of the study sample, case definition and classification of AMD [20–22]. Another important feature of AMD is that its prevalence is likely to be associated with geographic factors. In the most recent global geo–epidemiology analysis of AMD, both latitude and longitude were inversely correlated with AMD prevalence, providing a new clue to study the geographic distribution of AMD [15].

Until recently, there were no systematic estimates of AMD prevalence in China. With that said, the sheer volume of data available on the prevalence of AMD in Chinese bibliographical databases makes it possible to summarise the prevalence and burden of AMD from a modelling perspective [23,24]. Moreover, the large territory area with great variation of latitude and longitude in China provides a good opportunity to explore the influence of geographic factors within the same country. In this study, we undertook a comprehensive systematic review, in both Chinese and English databases, to retrieve population–based studies of AMD prevalence in China from 1990 onwards. Based on the existing evidence, we estimated and projected the prevalence and burden of AMD and its sub–types. The aims of this study were to 1) ascertain the AMD prevalence in China by using epidemiological modelling; 2) estimate and project the overall prevalence and number of people living with AMD at the national level from 1990 to 2050; 3) estimate the regional prevalence and number of people with AMD from 2000 to 2010.

## METHODS

### Systematic review

For developing epidemiological models to estimate the prevalence of AMD and its subtypes in the general population, a systematic review was conducted by two independent reviewers (PS and YD) in accordance with the Preferred Reporting Items for Systematic reviews and Meta–Analyses (PRISMA) guidelines and the Guidelines for Accurate and Transparent Health Estimates Reporting (GATHER) statement [25,26]. To ensure that all possible informative studies are included, a comprehensive literature search (title, abstract and keywords) was conducted in order to identify relevant studies. First, three Chinese bibliographic databases and three English bibliographic databases were searched from inception to 17 September 2016. These were the China National Knowledge Infrastructure (CNKI), Wanfang, Chinese Biomedicine Literature Database (CBM–SinoMed), PubMed, Embase and Medline. The source of studies in the three Chinese databases included journal articles, abstracts, dissertations and conference proceedings, whereas those in the three English databases included journal articles only. A combination of search terms for prevalence (prevalence, incidence, mortality, morbidity, epidemiology), AMD (age–related macular degeneration, age–related maculopathy, retina* macula* age related degeneration, retina* macular degeneration, macular degeneration) and China (China, Chinese, Hong Kong, Macau, Taiwan) was adopted for the comprehensive search. The final search strategy is presented in Table S1 in **Online Supplementary Document(Online Supplementary Document)**. Note that the search strategy for the different bibliographic databases was slightly different based on the database’s specific search features. Snowball searching of reference lists of publications retrieved in the first step was then conducted to further identify studies of interest. Only studies published since 1990 were retrieved and no language restrictions were imposed.

Only population–based studies that quantified the prevalence of AMD were included in this study. This is because studies conducted at institutional sites tend to have poor representativeness of the surrounding general population, especially for affected people living in poor and rural areas where access to health is not universal. Studies that relied on self–reported diagnosis were also excluded, due to recall bias. Studies that only reported the number of eyes affected by AMD, rather than the number of affected individuals, were also excluded because no prevalence of AMD could be derived from such studies. Duplicate publications of the same study were compared and the study providing more details was retained. Some additional criteria were also applied to ensure the quality of included studies. The detailed selection criteria are shown in **Table 1**.

**Table 1:** Selection criteria of studies in the systematic review

Inclusion criteria
1) Community–based study of AMD in China (including Hong Kong, Macao and Taiwan)
2) Studies conducted to examine the epidemiology of AMD
3) Studies reported numerical prevalence measure of AMD
**Exclusion criteria**
1) Multiple publications of the same study
2) Studies with no professional assessment methods or relied on self–reported diagnoses
3) Studies that were conducted in a population with characteristics that were clearly unrepresentative, eg, visual impaired population, diabetes population
4) Studies with inconsistencies between reported methods and presented results

Before reviewing the retrieved records, duplicates were removed manually. Records were screened for relevance in two stages: screening of titles and abstracts followed by the retrieval and check of full–text articles. All non–English or non–Chinese language documents were reviewed after translation into English by Google Translate. For studies that fulfilled the criteria, three main categories of data were extracted: characteristics of the study, characteristics of the investigated population, and prevalence estimates of AMD and its subtypes. The data extraction tables were pilot tested on ten randomly selected included studies and refined accordingly before the final extraction.

The final data extraction table included:

1. Characteristics of the study: authors, publication year, study setting, year of survey, sampling method, study design (cross–sectional or cohort), AMD assessment method, and AMD grading system;
2. Characteristics of the investigated population: number of the sample, population type (urban, rural or mixed), gender (male, female or mixed), and age (age range, mean or median age, or midpoint of the age range);
3. Prevalence data: number of people with AMD and the number of participants who had been tested, by age group, gender, setting and AMD subtype where available.

The geographic indicators of interest (latitude, longitude and average annual insolation) were assigned to each study accordingly. The latitude and longitude data were obtained using Google Maps GPS coordinates (http://www.gps–coordinates.net/). The average annual insolation data (ie, the amount of solar radiation incident on the surface of the earth) on the horizontal surface, expressed in kWh/m^2^/d, was obtained from the National Aeronautics and Space Administration (NASA) Atmospheric Science Data Centre (http://eosweb.larc.nasa.gov/sse/). When study settings were defined as larger regions, such as at province, or regional levels, the mean centre point of the setting was calculated and the corresponding geographic data of the centre point was used. Studies that reported raw prevalence data in more than one geographic area (eg, a single study presented prevalence of AMD for three different cities) were recorded separately for each geographic area. For studies that reported aggregated AMD prevalence data for different geographic areas, the average geographic data of the different areas were calculated and recorded. For studies with missing data of survey year, three years were subtracted from the published year to impute the survey year, which was based on the average time from survey to publication in studies with available data. In studies where censoring age groups were reported, eg, older than 80 years, the missing age band was taken as the same width as other age groups in the same study.

The classification systems used to define AMD and its subtypes include the Wisconsin age–related maculopathy system (WARMGS) [27], the International Classification and Grading system (IC) [28], the Clinical Age–Related Maculopathy Grading System (CARMS) [29], and the “Age–related Macular Degeneration Clinical Diagnosis Standard” proposed by the China Medical Association in 1986 (CMA1986) [30]. For studies adopting different classification systems, the prevalence of any AMD, early AMD, late AMD, which included GA and NVAMD, was extracted or calculated (if necessary) separately according to the definitions below:

1. Early AMD: any soft drusen (distinct or indistinct) and pigmentary abnormalities, or large soft drusen 125 μm or more in diameter with a large drusen area (>500 μm diameter circle) or large soft indistinct drusen in the absence of signs of late–stage disease;
2. Late AMD: the presence of geographic atrophy or pigment epithelial detachment, subretinal haemorrhage or visible subretinal new vessel, or subretinal fibrous scar or laser treatment scar.

### Statistical analysis

Due to high heterogeneity between studies that reported prevalence rates for any AMD, early AMD, late AMD, GA and NVAMD (Table S2 in **Online Supplementary Document(Online Supplementary Document)**), random–effect models were adopted throughout the analysis. In the data extraction process, data were stratified by age, gender and setting. Some studies provided more than one data point. To take this hierarchical data structure into account, a multilevel mixed–effect meta–regression was conducted [31,32]. Given that: 

Then, the binomial distribution of prevalence rates was transferred to the normal distribution by using logit link: 

Estimates were back transformed and expressed as conventional prevalence: 

To develop the overall “envelope” of AMD cases in China from 1990 to 2015, five models were first developed to establish the prevalence of any AMD, early AMD, late AMD, GA and NVAMD as a function of age: 

Thus, the prevalence of AMD is: 

The total number of AMD cases (“envelope”) in China was calculated by multiplying the age–specific prevalence of AMD for each 5–year age group estimated in the above models with the corresponding 5–year population subgroups in China, available from the United Nations Population Division (UNPD) [19]. This was performed for any AMD, early AMD, late AMD, GA and NVAMD separately in the years 1990, 2000, 2010 and 2015.

To investigate whether study–level demographic and geographic factors might affect the prevalence of AMD, variables of interest were added into the multilevel mixed–effect meta–regression to test the significance [33]. As a rule, at least seven data points should be available for each variable [34]. These variables included gender, setting, latitude, longitude and average annual insolation. Investigation year was also tested so as to assess if there were any significant time trends. All variables that individually associated AMD prevalence in univariable analyses were included in the subsequent multivariable regression model, where variables that were not statistically significant were removed, starting from the one with the highest p value.

For our projection to the year 2050, age–specific prevalence rates of AMD were assumed to be constant over the next 33 years, the number of individuals with AMD from 2020 to 2050 was calculated by multiplying the age–specific prevalence rates to the UNDP Prospects data [19].

Based on the final multivariable regression models that take the effects of demographic and geographic factors into consideration, the estimated national population with AMD was distributed into six geographical regions, namely, East China, North China, Northeast China, Northwest China, South Central China, Southwest China (**Table 2**) [35–37]. This method was initially proposed by the Child Health Epidemiology Reference Group (CHERG), and has, since, been adopted widely in disease burden research [38–40]. First, AMD prevalence in each geographic region was calculated, based on the final regression equation. Second, the regional population with AMD was estimated by multiplying the regional AMD prevalence and corresponding population for the years 2000 and 2010, where regional population data were available from the fifth and sixth census [36,37]. Finally, the regional population of AMD was adjusted to fit the national AMD “envelope”.

**Table 2:** The six geographical regions in China

Region	Included provinces
North China	Beijing Municipality, Hebei province, Inner Mongolia Autonomous Region, Shanxi province, Tianjin Municipality
Northeast China	Heilongjiang province, Jilin province, Liaoning province;
East China	Anhui province, Fujian province, Jiangsu province, Jiangxi province, Shandong province, Shanghai Municipality, Zhejiang province
South Central China	Guangdong province, Guangxi Zhuang Autonomous Region, Hainan province, Henan province, Hubei province, Hunan province
Southwest China	Chongqing Municipality, Guizhou province, Sichuan province, Tibet Autonomous Region, Yunnan province
Northwest China	Gansu province, Ningxia Hui Autonomous Region, Qinghai province, Shaanxi province, Xinjiang Uyghur Autonomous Region

Non–dichotomous variables were analysed as continuous. A two–sided p value less than 0.05 was regarded as statistically significant for all analyses. All statistical analyses were performed in R Studio (version 1.0.136) built on R (version 3.3.0). All included studies in the analysis were mapped by ArcGIS software (Version 10.1). The China base map was obtained as a shapefile from the Global Administrative Areas (GADM) database (GADM, 2015, version 2.0; www.gadm.org).

## RESULTS

### Summary of systematic review

**Figure 1:**
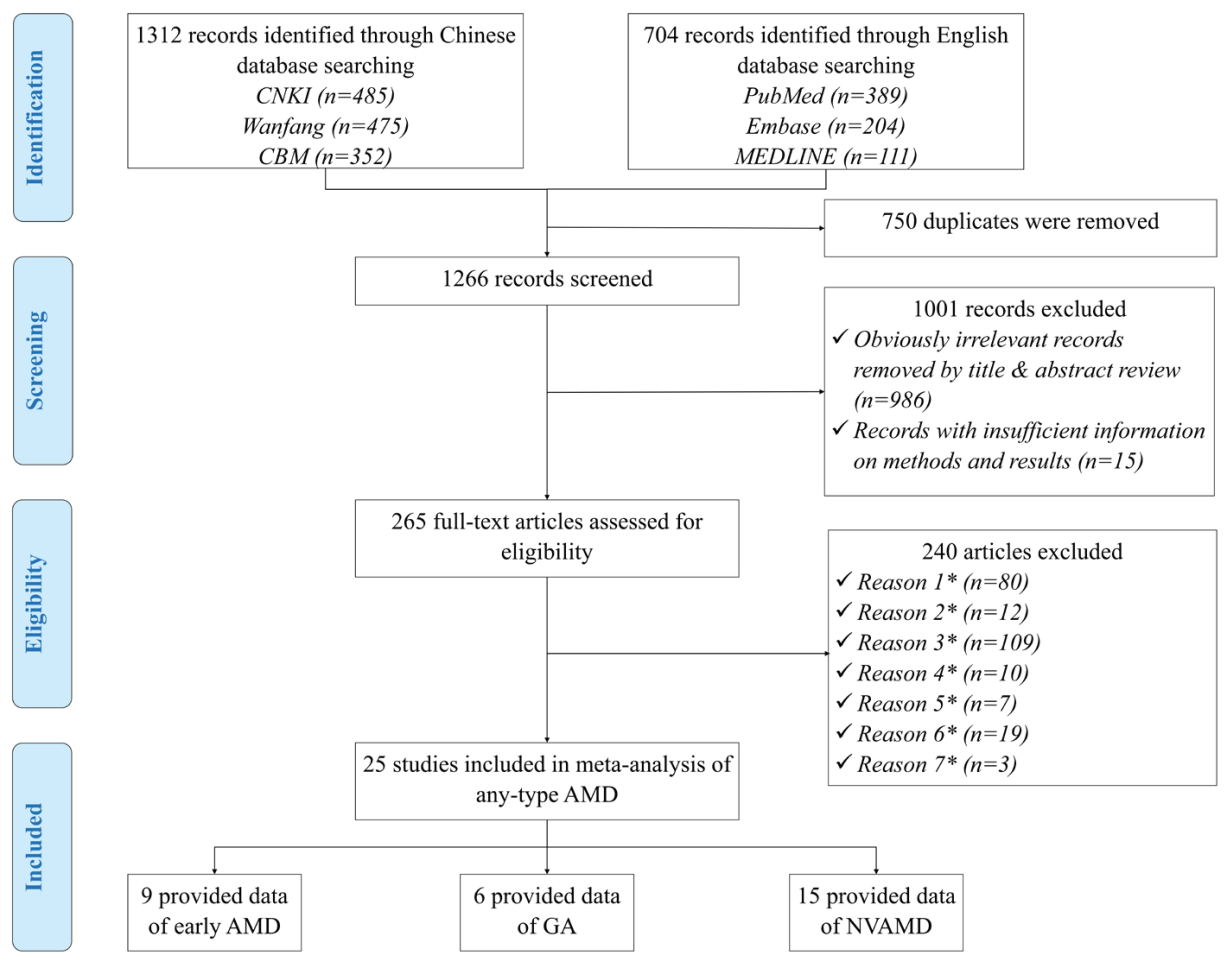
Systematic review flow diagram. Note: *Reason 1 – Studies that were not population–based; *Reason 2 – Studies that were not based in China; *Reason 3 – Papers with no numerical prevalence measure of AMD; *Reason 4 – Studies that had no professional assessment methods or relied on self–reported diagnoses; *Reason 5 – Studies that were conducted in a population with unrepresentative characteristics; *Reason 6 – Multiple publications of the same study; *Reason 7 – Papers with inconsistency between reported methods and presented results.

**Figure 1** Shows the process of systematic review for studies included in the final meta–analysis. In brief, the initial search identified 2016 citations. After removing 750 duplications, 986 apparently irrelevant citations by title and abstract review, and 15 citations with no sufficient information on methods and results, 265 papers were reviewed at the full–text level to assess their eligibility. Ultimately, 25 AMD prevalence studies were included in the final analysis.

**Figure 2:**
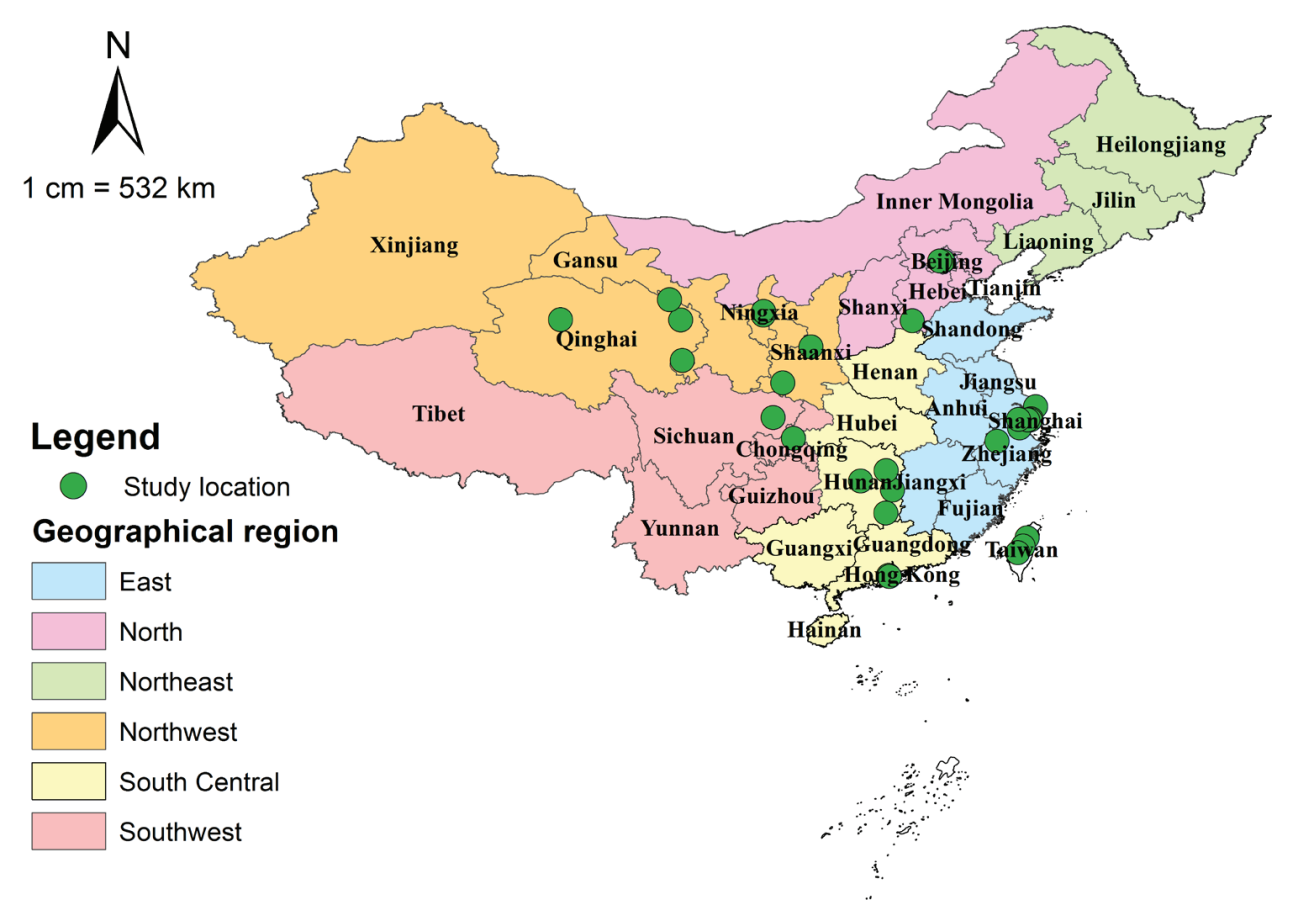
Geographic distribution of the included studies in China.

A full list of included studies is shown in Table S3 in **Online Supplementary Document(Online Supplementary Document)**, the included data involved 3016 AMD cases in a total of 43 420 examined individuals. **Table 3** shows the main characteristics of the studies, and the detailed characteristics of every study can be found in Table S4 in **Online Supplementary Document(Online Supplementary Document)**. All included studies were cross–sectional studies that assessed AMD by using fundus imaging. Almost half of the retained studies were published in the past six years (44.0%), with CMA1986 the most widely adopted grading system (48.0%), followed by WARMGS (24.0%) and CARMS (20.0%). The geographic distribution of the 25 included studies is demonstrated in **Figure 2**.

**Table 3:** Main characteristics of the included prevalence studies (n = 25)

Characteristics of study	Number of studies (%)
**Year published:**	
1990–1999	7 (28.0)
2000–2009	7 (28.0)
2010–2016	11 (44.0)
**Setting:**	
Urban	7 (28.0)
Rural	9 (36.0)
Mixed	9 (36.0)
**Sample size:**	
600–1000	5 (20.0)
1001–2000	5 (20.0)
2001–3000	7 (28.0)
3001–5000	4 (16.0)
5001–8000	4 (16.0)
**Grading system:**	
CMA1986*	12 (48.0)
WARMGS†	6 (24.0)
CARMS‡	5 (20.0)
IC§	1 (4.0)
Other‖	1 (4.0)

*CAM 1986 – the “Age–related Macular Degeneration Clinical Diagnosis Standard” proposed by the China Medical Association in 1986.

†WARMGS – the Wisconsin age–related maculopathy system.

‡CARMS, the Clinical Age–Related Maculopathy Grading System.

§IC, the International Classification and Grading system.

‖Other, definition in the “Ophthalmology” (7^th^ version).

### Age–specific prevalence of AMD

**Figure 3:**
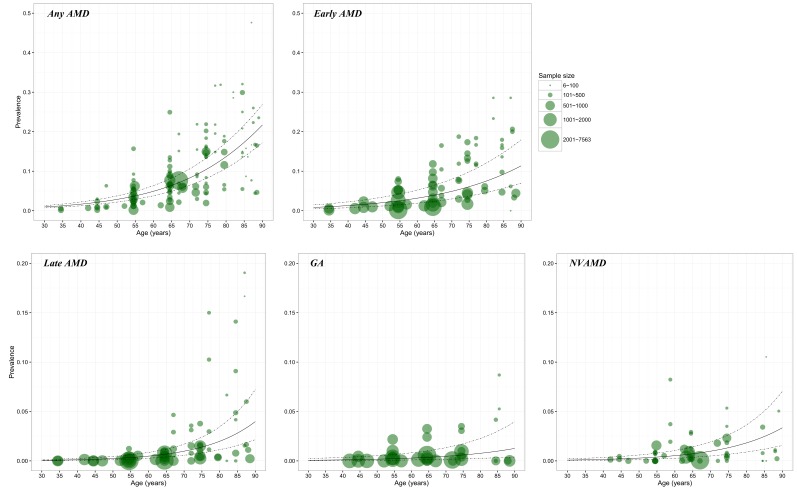
Prevalence of age–related macular degeneration (AMD) and its subtypes by age in retained studies. Note: The size of each bubble is proportional to the sample size. There were 124 data points for constructing the relation between prevalence and age for any AMD, 67 for early AMD, 67 for late AMD, 35 for geographic atrophy (GA) and 54 for neovascular AMD (NVAMD).

In each model (**Figure 3**), a substantial number of data points were available for constructing the relationship between AMD prevalence and age. The age spectrum ranged from around 35 years to less than 90 years. However, for GA and NVAMD, few data points were available at younger ages (30–40 years). In this study, to ensure that the estimated prevalence was comparable, the lower bound of age range was set as 45 years and the upper bound as 89 years where data were available for model construction at all AMD subtype groups.

**Figure 4:**
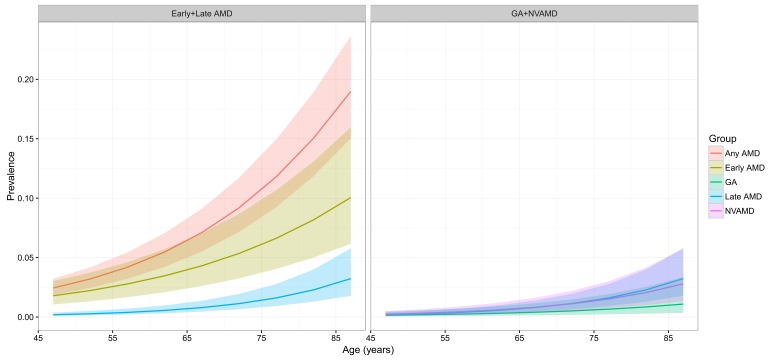
Estimated age–specific prevalence of age–related macular degeneration (AMD) and its subtypes in China, with 95% confidence intervals. GA – geographic atrophy, NVAMD – neovascular AMD.

The estimated age–specific prevalence of any AMD, early AMD, late AMD, GA and NVAMD is shown in **Figure 4** and **Table 4**. The prevalence of any AMD ranged from 2.44% (95% CI = 1.85–3.22) in people aged 45–49 years to 18.98% (95% CI = 15.05–23.66) in people aged 85–89 years. Prevalence of early AMD ranged from 1.79% (95% CI = 1.05–3.02) to 10.05% (95% CI = 6.17–15.97), and, in the case of late AMD, from 0.38% (95% CI = 0.16–0.97) to 3.88% (95% CI = 1.68–9.13). In late AMD, the prevalence of GA was 0.15% (95% CI = 0.05–0.47) in people aged 45–49 years and 1.09% (95% CI = 0.35–3.36) in those aged 85–89 years, and the prevalence of NVAMD ranged between 0.24% (95% CI = 0.11–0.50) and 2.79% (95% CI = 1.33–5.77).

**Table 4:** Estimated age–specific prevalence (% and 95% confidence interval) of age–related macular degeneration (AMD) and its subtypes in China

Age (years)	Any AMD	Early AMD	Late AMD	GA	NVAMD
45–49 y	2.44	1.79	0.38	0.15	0.24
	(1.85–3.22)	(1.05–3.02)	(0.16–0.97)	(0.05–0.47)	(0.11–0.50)
50–54 y	3.21	2.23	0.51	0.19	0.32
	(2.45–4.19)	(1.32–3.74)	(0.22–1.24)	(0.06–0.58)	(0.16–0.67)
55–59 y	4.20	2.78	0.68	0.24	0.44
	(3.22–5.45)	(1.67–4.62)	(0.30–1.60)	(0.08–0.72)	(0.22–0.89)
60–64 y	5.47	3.47	0.91	0.31	0.60
	(4.23–7.06)	(2.09–5.71)	(0.41–2.09)	(0.11–0.90)	(0.30–1.19)
65–69 y	7.11	4.31	1.22	0.40	0.82
	(5.52–9.12)	(2.61–7.05)	(0.55–2.76)	(0.14–1.15)	(0.41–1.60)
70–74 y	9.20	5.36	1.63	0.52	1.11
	(7.17–11.72)	(3.26–8.68)	(0.74–3.67)	(0.18–1.48)	(0.56–2.19)
75–79 y	11.81	6.63	2.18	0.66	1.52
	(9.26–14.96)	(4.05–10.68)	(0.98–4.94)	(0.23–1.93)	(0.76–3.01)
80–84 y	15.05	8.18	2.91	0.85	2.06
	(11.85–18.92)	(5.01–13.09)	(1.29–6.70)	(0.28–2.54)	(1.01–4.16)
85–89 y	18.98	10.05	3.88	1.09	2.79
	(15.05–23.66)	(6.17–15.97)	(1.68–9.13)	(0.35–3.36)	(1.33–5.77)

GA – geographic atrophy, NVAMD – neovascular AMD

### National population affected with AMD from 1990 to 2015

**Figure 5:**
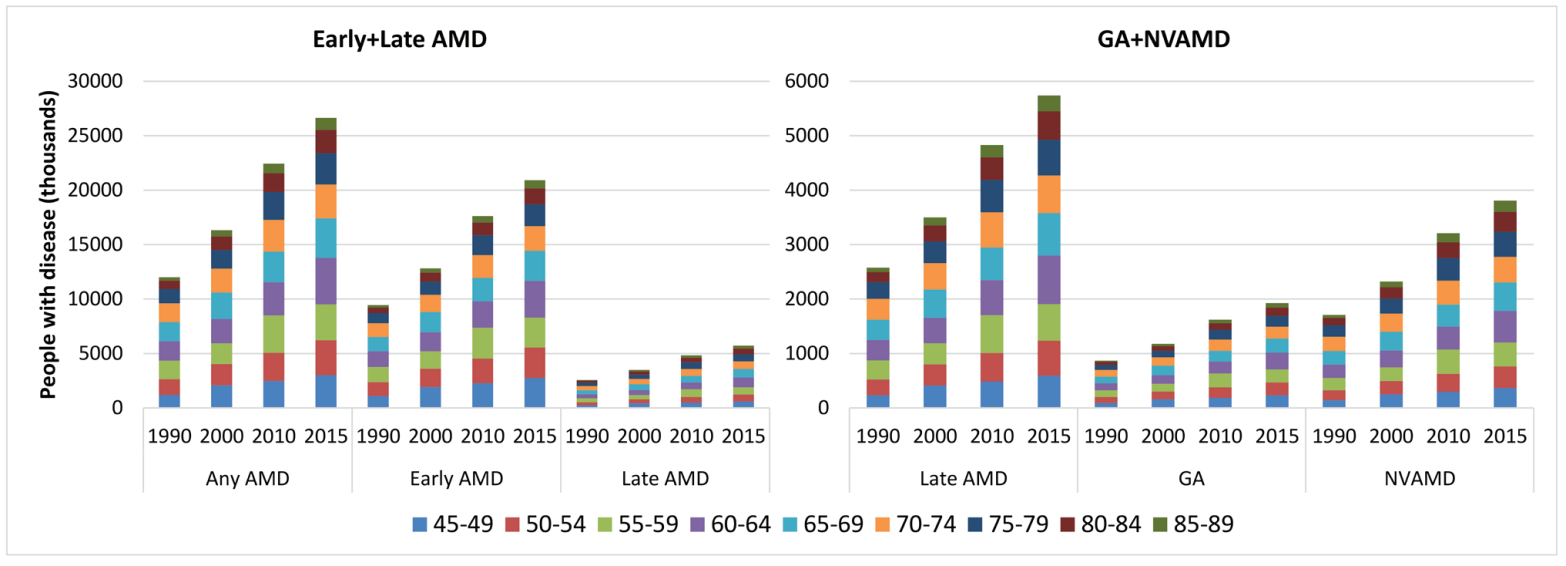
Estimate of the national number of people with age–related macular degeneration (AMD) and contributing age groups in China from 1990 to 2015, by AMD type. GA – geographic atrophy, NVAMD – neovascular AMD.

By applying the age–specific prevalence of AMD to the national population in 1990, 2000, 2010 and 2015, the number of people living with AMD in China was estimated (Table S5 in **Online Supplementary Document(Online Supplementary Document)**). During this period, the national prevalence of any AMD slightly decreased by 0.41%, from 5.26% (95% CI = 4.07–6.76) in 1990 to 5.24% (95% CI = 4.05–6.73) in 2015. This declining trend was also witnessed in early AMD and late AMD, with decreasing rates of 0.50% and 0.07% respectively. In late AMD, GA also showed a decreasing trend within this time frame, whereas the prevalence of NVAMD increased slightly (**Table 5**). Despite this decreasing prevalence trend during 1990–2015, the overall number of people with any AMD or its subtypes all increased dramatically due to the rapidly ageing population. The national number of people with any AMD increased by 121.80%, from 12.01 million (95% CI = 9.29–15.46) in 1990 to 26.65 million (95% CI = 20.62–34.27) in 2015. Within the same period, the number of people with early AMD increased from 9.44 million (95% CI = 7.74–11.15) to 20.91 million (95% CI = 17.16–24.68), and those with late AMD rose from 2.58 million (95% CI = 1.56–4.30) to 5.74 million (95% CI = 3.46–9.59), which yielded increasing rates of 121.60% and 122.55% respectively. In late AMD, increase in the number of people living with GA was similar to those with NVAMD (121.99% vs 122.84%), which ranged from 0.87 million (95% CI = 0.40–1.83) to 1.93 million (95% CI = 0.89–4.08), and 1.71 million (95% CI = 1.16–2.47) to 3.81 million (95% CI = 2.57–5.51) throughout this time frame respectively (**Table 5**). In 2015, the age group that contributed the most cases of any AMD, early AMD, late AMD, GA and NVAMD was 60–64 years (**Figure 5**).

**Table 5:** Estimated prevalence and number of people living with age–related macular degeneration (AMD) in China from 1990 to 2015, by AMD type

AMD type	Prevalence of AMD (%, 95% CI)		Number of people with AMD (million, 95% CI)		Rate of change (%, 1990–2015)	
**1990**	**2015**	**1990**	**2015**	**prevalence**	**AMD cases**
Any AMD	5.26	5.24	12.01	26.65	–0.41	121.80
	(4.07–6.76)	(4.05–6.73)	(9.29–15.46)	(20.62–34.27)		
Early AMD	4.13	4.11	9.44	20.91	–0.50	121.60
	(3.39–4.88)	(3.37–4.85)	(7.74–11.15)	(17.16–24.68)		
Late AMD	1.13	1.13	2.58	5.74	–0.07	122.55
	(0.68–1.88)	(0.68–1.88)	(1.56–4.30)	(3.46–9.59)		
GA	0.38	0.38	0.87	1.93	–0.33	121.99
	(0.17–0.80)	(0.17–0.80)	(0.40–1.83)	(0.89–4.08)		
NVAMD	0.75	0.75	1.71	3.81	0.05	122.84
	(0.51–1.08)	(0.51–1.08)	(1.16–2.47)	(2.57–5.51)		

GA – geographic atrophy, NVAMD – neovascular AMD

### Effects of demographic and geographic factors on the prevalence of AMD

Findings from the univariable meta–regression analyses (Table S6 in **Online Supplementary Document(Online Supplementary Document)**) showed that age, setting and latitude were significantly associated with the prevalence of any AMD. For early AMD, age, gender, setting and latitude also had a significant influence on the prevalence. For late AMD, age and latitude were found to be significantly associated with the prevalence. However, in late AMD, only age was found to be significantly associated with the prevalence of GA, and age, gender, latitude and insolation were significantly associated with the prevalence of NVAMD. For any AMD and all subtypes, the investigation year was identified to have no influence on prevalence rates and increased age was the only constantly significant risk factor.

Although most studies provided multiple data points of prevalence rates, these data were mainly stratified by age groups. For AMD subtype groups (early AMD, late AMD, GA and NVAMD), and after controlling the difference of age structures, insufficient data were available for conducting multivariable meta–regression that simultaneously included all statistically significant factors identified in the univariable analyses. Thus, here the multivariable regression model was only conducted and reported for any AMD. The formula generated from the multivariable regression is shown below: 

Where *p* indicates the prevalence of any AMD; *setting_rural_* = 1 for rural setting and = 0 otherwise; *setting_urban_* = 1 for urban setting and = 0 otherwise; *latitude* refers to the absolute value of latitude.

### Projection of national population affected with AMD from 2020 to 2050

No secular trend of the prevalence of any AMD, early AMD, late AMD, GA and NVAMD was observed in the included studies, thus age–specific prevalence was assumed as constant for the projection analysis. By applying the age–specific prevalence of AMD to the national population in 2020, 2030, 2040 and 2050, the number of people with AMD was estimated (Tables S5 in **Online Supplementary Document(Online Supplementary Document)**). Unlike the slightly fluctuating trend of AMD prevalence during 1990 to 2015, the prevalence rates of all subtypes of AMD will increase notably during 2020 and 2050. In 2020, the prevalence of any AMD will be 5.39% (95% CI = 4.18–6.93) and is expected to increase by 41.66%, reaching to 7.64% (95% CI = 5.96–9.73) in 2050. Among all subtypes of AMD, NVAMD will show the greatest increasing rate of 57.48%, from 0.78% (95% CI = 0.52–1.12) in 2020 to 1.22% (95% CI = 0.83–1.75) in 2050, whereas the increasing rate of early AMD will be the smallest (38.45%), from 4.23% (95% CI = 3.47–4.99) to 5.21% (95% CI = 4.31–6.08) during this period (**Table 6**).

**Table 6:** Projected prevalence and number of people living with age–related macular degeneration (AMD) in China from 2020 to 2050, by AMD type

AMD type	Prevalence of AMD (%, 95% CI)		Number of people with AMD (million, 95% CI)		Rate of change (%, 2020–2050)	
**2020**	**2050**	**2020**	**2050**	**prevalence**	**AMD cases**
Any AMD	5.39	7.64	31.23	55.19	41.66	76.72
	(4.18–6.93)	(5.96–9.73)	(24.18–40.14)	(43.04–70.30)		
Early AMD	4.23	5.85	24.47	42.26	38.45	72.71
	(3.47–4.99)	(4.87–6.79)	(20.10–28.87)	(35.15–49.05)		
Late AMD	1.17	1.79	6.76	12.92	53.28	91.21
	(0.70–1.95)	(1.09–2.94)	(4.08–11.28)	(7.89–21.26)		
GA	0.39	0.57	2.26	4.09	44.92	80.78
	(0.18–0.83)	(0.26–1.19)	(1.04–4.78)	(1.89–8.59)		
NVAMD	0.78	1.22	4.50	8.84	57.48	96.45
	(0.52–1.12)	(0.83–1.75)	(3.04–6.50)	(6.00–12.66)		

GA – geographic atrophy, NVAMD – neovascular AMD

**Figure 6:**
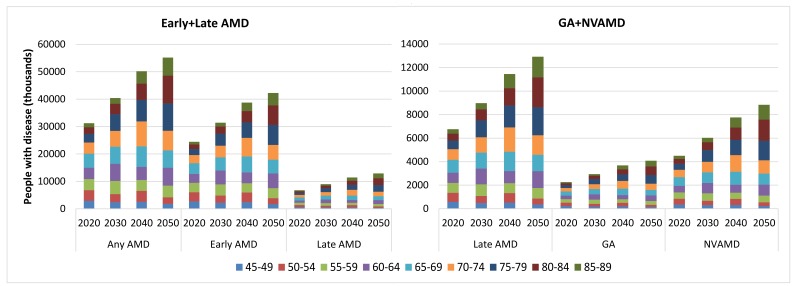
Projection of the national number of people with age–related macular degeneration (AMD) and contributing age groups in China from 2020 to 2050, by AMD type. GA – geographic atrophy, NVAMD – neovascular AMD.

From 2020 to 2050, the number of cases of any AMD in China will rise by 76.72%, from 31.23 million (95% CI = 24.18–40.14) to 55.19 million (95% CI = 43.04–70.30). The increasing rate of late AMD cases will be greater than early AMD cases (91.12% vs 72.70%), with the number of people affected by early AMD increasing from 24.47 million (95% CI = 20.10–28.87) in 2020 to 42.26 million (95% CI = 35.15–49.05) in 2050, and those affected by late AMD from 6.76 million (95% CI = 4.08–11.28) to 12.92 million (95% CI = 7.89–21.26). In late AMD, the number of people with GA will increase by 80.78%, from 2.26 million (95% CI = 1.04–4.78) in 2020, to 4.09 million (95% CI = 1.89–8.59) in 2050. Furthermore, the number of those with NVAMD will grow even further (96.45%), from 4.50 million (95% CI = 3.04–6.50) to 8.84 million (95% CI = 6.00–12.66) (**Table 6**). From 2020 to 2050, the age groups to contribute the most cases will shift from 65–69 years to 80–84 years for any AMD, late AMD, GA and NVAMD, and from 65–69 years to 75–79 years for early AMD (**Figure 6**).

### Regional population affected with AMD from 2000 to 2010

The total number of AMD cases in China in 2000 and 2010 was distributed across the six geographical regions according to the final multivariable model that took into account three main factors: age, setting and latitude. In 2000, the national prevalence of AMD in China was 5.16% (95% CI = 3.99–6.64), with the regional prevalence estimates ranging from 2.69% (95% CI = 1.67–4.29) in North–East China to 6.64% (95% CI = 5.12–8.52) in South Central China. In 2010, the prevalence was still the highest in South Central China (6.74% [95% CI = 5.20–8.65]) and the lowest in North–East China (2.65% [95% CI = 1.66–4.20]), with the overall prevalence in Chinese population increasing to the level of 5.24% (95% CI = 4.05–6.73). During 2000 to 2010, the overall prevalence of AMD increased by 1.44%, and the most marked increase was in Southwest China (6.51%) while the prevalence rate of AMD declined by 1.37% in North–East China (**Table 7**).

**Table 7:** Estimated prevalence and number of people living with any age–related macular degeneration (AMD) in China in the years 2000 and 2010, by geographical region

Region	Prevalence of AMD (%, 95% CI)		Number of people with AMD (million, 95% CI)		Rate of change (%, 2000–2010)	
**2000**	**2010**	**2000**	**2010**	**prevalence**	**AMD cases**
North China	3.28	3.36	1.23	1.82	2.40	48.18
	(2.35–4.55)	(2.42–4.64)	(0.88–1.70)	(1.31–2.51)		
North–East China	2.69	2.65	0.76	1.11	–1.37	46.70
	(1.67–4.29)	(1.66–4.20)	(0.47–1.21)	(0.69–1.76)		
East China	5.46	5.57	5.32	7.33	1.99	37.67
	(4.39–6.74)	(4.49–6.86)	(4.29–6.57)	(5.91–9.04)		
South Central China	6.64	6.74	5.50	7.52	1.54	36.74
	(5.12–8.52)	(5.20–8.65)	(4.24–7.05)	(5.80–9.64)		
South–West China	5.68	6.05	2.85	3.70	6.51	29.97
	(4.50–7.11)	(4.81–7.56)	(2.26–3.56)	(2.94–4.62)		
North–West China	3.29	3.40	0.66	0.95	3.23	44.65
	(2.44–4.43)	(2.53–4.55)	(0.49–0.89)	(0.71–1.28)		
China	5.16	5.24	16.31	22.43	1.44	37.50
	(3.99–6.64)	(4.05–6.73)	(12.62–20.99)	(17.36–28.85)		

**Figure 7:**
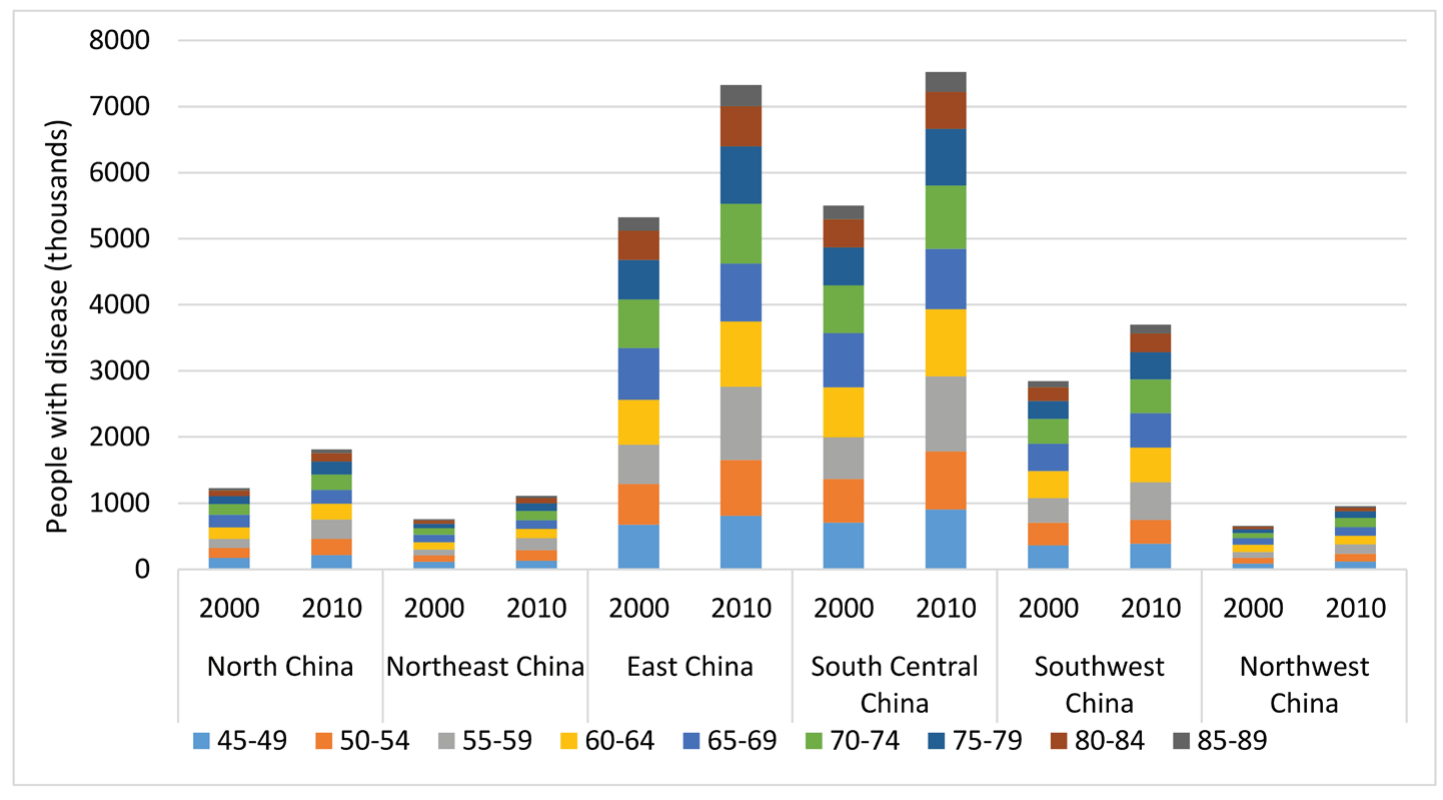
Estimate of the regional number of people with age–related macular degeneration (AMD) and contributing age groups in China in the years 2000 and 2010.

Estimates of the number of people living with AMD in different regions are shown in **Table 7** and **Figure 7**. With the ageing trend of the Chinese population, the total number of people living with AMD in China increased by 37.50%, from 16.31million (95% CI = 12.62–20.99) in 2000 to 22.43 million (95% CI = 17.36–28.85) in 2010. In 2000, more than one–third (33.72%) of Chinese AMD cases were found living in South Central China (5.50 million, 95% CI = 4.24–7.05) and only 4.05% were in North–West China (0.66 million, 95% CI = 0.49–0.89). In 2010, this distribution of AMD cases remained the same across the six geographical regions, with most (33.53%) of the AMD cases in South Central China (7.52 million, 95% CI = 4.24–7.05) and the least (4.24%) in North–West China (0.95 million, 95% CI = 0.71–1.28). From 2000 to 2010, the most striking increases in the number of AMD cases were in North China (48.64%) and North–East China (47.06%), and the least in South–West China (29.97%). Throughout this decade, the age groups that contributed the most AMD cases shifted from 55–59 years to 60–64 years in all of the six regions.

## DISCUSSION

In this systematic review and meta–analysis, data–driven estimates and projections of AMD prevalence and burden in China were presented, both at the national level and at the regional levels. The results from this synthesised population–based data show that the burden of AMD in China is substantial. From 1990 to 2015, the prevalence of AMD fluctuated at around 5.2%, which translates to a total of 26.65 million affected individuals in 2015. By 2050, prevalence of AMDis expected to increase to 7.64%, with the corresponding number of affected individuals being 55.19 million. Substantial regional variation was found across the country, with AMD prevalence being the highest in South Central China and the lowest in the North–East. In terms of the total number of AMD cases, the greatest burden was in the South Central area, and the smallest in North–West China.

To the best of our knowledge, this study is the first attempt to estimate the prevalence and the burden of AMD in China and to make future projections. The comprehensive search strategies and strict inclusion and exclusion criteria ensured a well–designed analysis. Furthermore, the current study provided estimates of the prevalence and the number of affected AMD cases by AMD subtype, with this additional information being of particular clinical and public health relevance. Indeed, such information offers valuable, detailed insights into the burden of AMD in China. Critically, this study used the best available data to portray a complete picture of the public health burden of AMD in different regions. Therefore, it can serve as the basis for health policy making and resource allocation for AMD prevention and treatment initiatives. From a global perspective, this study complements the most recent Global AMD study, where insufficient data were available for national estimates and projections [3].

However, this study is not free from limitations. First, significant heterogeneity existed between all of the included studies, despite the strict inclusion and exclusion criteria applied. Like any meta–analysis, the findings of this study are only as good as the included primary investigations. Included studies did not come from across the country, thus the ability to generate provincial estimates of AMD prevalence and cases may be limited. Second, one NVAMD–like disease, polypoidal choroidal vasculopathy, is markedly more common in Asians [3,41,42], and taking into consideration that most population–based studies may have limited ability to distinguish between these two diseases (as suggested previously [3]), the prevalence and burden of NVAMD in the current study may be overestimated. In addition, as suggested by a previous meta–analysis, studies using fundus imaging with classifications, rather than the internationally recognised grading systems, are more likely to diagnose late AMD [13]. In this study, almost half of the included studies adopted the grading system proposed by the Chinese Medical Association, which may also have contributed to the peculiarly elevated prevalence rate of late AMD. A further point to raise is that only a limited set of variables were included and explored in the meta–regression analysis. This means that there could have also been further explanatory variables that may influence the presence of AMD. Moreover, the included demographic and geographic variables were mainly aggregate level data, and although efforts were made to extract data stratified by age, gender and location, the variation at the individual level may still be hidden. This may include smoking exposure, the habit of wearing sunglasses, and others. A further limitation of the study is that the estimates of regional prevalence and burden of AMD were based on the assumption that the pooled prevalence estimate for a specific region was homogeneous across all included provinces within this region, but this is quite unrealistic. Additionally, for regions that contributed only a few, or no actual AMD prevalence data points to the model, the model–based estimates may diverge quite considerably from the true prevalence. Finally, as reported in both previous reviews and substantiated in the current study, the prevalence of AMD and all its subtypes was stable over time [3,13]. Based on this assumption, the projections of the national prevalence and burden of AMD were actually based on the model–based age–specific prevalence and demographic changes during the next three decades. Thereby, the uncertainty of these projections may be largely dependent on the accuracy of age–specific prevalence model and the UNPD population projection. Bearing these limitations in mind, estimates presented in the current study should be interpreted judiciously.

In this study, the overall prevalence of AMD among the Chinese population was lower than the estimates in the Global AMD study, which reported an overall AMD prevalence of 6.86% in people living in Asia [3]. In the Global AMD study, the prevalence estimates for Asia were based on eleven studies conducted across Asia, among which, six came from south Asia (India, Singapore, and Thailand), three from China and two from Japan. Given the fact that AMD prevalence increases with decreasing latitude, as detected in both this study and a previous global geo–epidemiology study of AMD [15], it is not surprising that the overall prevalence of AMD in the current study is lower than that in Asia – as estimated by the Global AMD study. Moreover, the eleven studies in the Global AMD study were each published in the 21st century, whereas those included in this study distributed from 1990 to 2014. Although no secular trend of AMD was detected in either the Global AMD study or this study, the difference of the estimated AMD prevalence in these two studies can still be partly explained by the difference of ageing demographic structure [3,13,15].

In line with previous population–based investigations and synthesised analysis [3,14,43], this study confirms two common notions of AMD with strong evidence. First, AMD is a degenerative and progressive disease, with the prevalence of AMD dramatically increasing with age, and with age also found to be the only constant risk factor in the presence of any AMD and all its subtypes. Second, the prevalence of early AMD was found to be much higher than that of late AMD. This finding, however, should not be misinterpreted as late AMD contributing a smaller burden. Rather, most individuals with early AMD may not go on to develop the late–stage disease, and late AMD is a much more severe disease than early AMD [9,13]. In this study, the prevalence of late AMD in Chinese people in 2015 was found to be even higher than that of people living in Europe (1.13% vs 0.75), the continently highest prevalence of both early and late AMD as revealed by the Global AMD study [3]. In view of the large population size in China, this striking finding highlights an urgent need for action on the prevention and treatment of late AMD, given its clinical significance. Compared to GA, the group of NVAMD represents a larger burden in Chinese population because the prevalence and number of people with NVAMD were estimated as around twice higher than those of GA. This phenomenon has been reported in some individual investigations [44,45], although it has not been universally acknowledged [13]. This finding is still of particular importance for the secondary prevention, especially for NVAMD, whose progress to sight loss could be slowed considerably by current treatment approaches – such as the use of anti–vascular endothelial growth factor agents [46,47].

In this study, AMD was found to be more prevalent in urban populations than in rural populations, with possible explanations for this disparity being the difference in environments (eg, UV exposure), as well as lifestyles (eg, education, profession and level of physical activity). While it is not possible to say precisely what the determinants are, this study clearly shows that people living in rural areas with a self–sustained economy are less likely to be affected by AMD [48,49].

A gradient of decreasing prevalence of AMD was noted in increasing latitude, which suggests that the special climate and environmental factors in geographical areas approximating the equator may accelerate the development of AMD. One common hypothesis is that AMD is associated with the amount of insolation [15]. However, the indicator of average annual insolation was only found to be significantly associated with late AMD in this present study. There are two possible reasons for this. First, annual insolation data were averaged over a 22–year period (July 1983 – June 2005), which may represent a considerable time–lag [50,51]. Second, the relation between insolation and the prevalence of AMD may not be a monotone function, the global geo–epidemiology study of AMD revealed higher prevalence rates of AMD in locations with insolation ≤3 kWh/m^2^/d compared with those with insolation >3 kWh/m^2^/d [15]. Although this interesting relation was not studied further because of limited data availability, the negative relation between latitude and AMD prevalence is an interesting hypothesis to explore in future Chinese AMD epidemiological studies.

Male gender was indicated as a risk factor for early AMD and NVAMD in the univariable regression analysis of this study. This is in contrast to previous reviews and individual investigations of populations of European ancestry, where females were reported to have a higher risk of developing NVAMD [13,45,52]. However, this study is underpowered to further confirm the observed gender difference in multivariable regression analysis. In the multivariable analysis of the prevalence of any AMD, no evidence of gender difference was found after adjusting for *a priori* demographic and geographic variables. This finding is consistent with the previous Global AMD study [3].

Variation in AMD prevalence and burden was noted in different geographic locations in China. The variation was mainly driven by the different demographic structures and the intrinsic environmental characteristics of these regions. According to the estimates for the six regions, AMD epidemics continue to be concentrated in the most populous South Central China. Taken together, these findings are of a particular public health interest in national health service allocation. Based on this study, more epidemiological investigations are required in order to make the regional estimates of subtypes of AMD in the future.

This work has important implications both in academic and public health areas. Future epidemiological studies of AMD in China would benefit from greater standardisation and improved design, ideally adopting internationally recognised grading systems and presenting results for different subtypes. In addition, as AMD is a priority eye disease that may lead to severe visual impairment or even blindness, its potential burden on individuals and health systems is particularly large in resource–limited settings [1,2,17]. Thus, localised epidemiological surveys should be conducted in socio–economically disadvantaged provinces, such as Tibet. From the national perspective, the public health impact of AMD is not only limited to the number of people affected, but also brings about multiple diagnostic and treatment challenges arising from this condition [53,54]. It is prudent to address the importance of primary prevention, such as smoking cessation [11,55], lifestyle modification, antioxidant therapy [56], and the use of hats and sunglasses [57]. In the meanwhile, the treatment of NVAMD is already available (although rather expensive) [47,58,59]. Given the remarkable potential economic burden on the society, government efforts must be taken to ensure the availability of health services to address AMD from the points of diagnosis and treatment, and even prevention when available.

To conclude, this systematic review and meta–analysis provides the first comprehensive and up–to–date estimate of AMD prevalence and burden in China. The results from this study indicate that the burden of AMD is substantial in China, with great variance among different subtypes and geographic regions. In the next decade and beyond, the ageing demographic will make this burden even larger. Improved epidemiological studies are still needed to inform optimal implementation of eye care programmes in China.
