# Supplementary material for: The national and subnational prevalence and burden of age–related macular degeneration in China
Source: J Glob Health. 2017 Dec 2;7(2):020703. doi: 10.7189/jogh.07.020703 (PMC5735777; doi:10.7189/jogh.07.020703)
Supplement: Online Supplementary Document [file jogh-07-020703-s001.pdf]

Online supplementary document

Song et al. The national and subnational prevalence and burden of age–related macular degeneration in China

J Glob Health 2017;7:020703

Table S1. Search strategy to identify studies reporting the prevalence of age-related macular degeneration in China

CNKI
Access Date: 26 JUN 2016
Subject category: Medicine & Public Health
Sub-database: Journal, Featured journal, Doctoral dissertation, Master dissertation, Domestic conferences, International conferences
检索表达式：
(SU % '年龄相关黄斑变性' + '年龄相关性黄斑变性' + '老年黄斑变性'+ '老年性黄斑变性' + '年龄相关黄斑病变'+ '年龄相关性黄斑病变' + '老年黄斑病变'+ '老年性黄斑病变') AND (SU % '发病率' + '发生率' + '患病率'+ '罹患率' + '现患率'+ '死亡率' + '病死率'+ '流行' + '负担'+ '现况调查'+ '现况研究')
发表时间：从 1990-01-01 到 2016-06-26
Search Terms: (SU % 'nianlingxiangguanhuangbanbianxing' + 'nianlingxiangguanxinghuangbanbianxing' + 'laonianhuangbanbianxing'+ 'laonianxinghuangbanbianxing' + 'nianlingxianghguanhuangbanbingbian'+ 'nianlingxiangguanxinghuangbanbingbian' + 'laonianhuangbanbingbian'+ 'laonianxinghuangbanbingbian') AND (SU % 'fabinglv' + 'fashenglv' + 'huanbinglv'+ 'lihuanlv' + 'xianhuanlv'+ 'siwanglv' + 'bingsilv'+ 'liuxing' + 'fudan'+ 'xiankuangdiaocha'+ 'xiankuangyanjiu')
Published time：From 01/01/1990 to 26/06/2016

Wanfang
Access Date: 26 Jun 2016
Sub-database: Journal articles, Dissertations, Conference articles, Foreign journals, Foreign conferences
检索表达式：(主题:(年龄相关黄斑变性) + 主题:(年龄相关性黄斑变性) + 主题:(老年黄斑变性) + 主题:(老年性黄斑变性) + 主题:(年龄相关黄斑病变) + 主题:(年龄相关性黄斑病变) + 主题:(老年黄斑病变) + 主题:(老年性黄斑病变)) * (主题:(发病率) + 主题:(发生率) + 主题:(患病率) +主题:(罹患率) + 主题:(现患率) + 主题:(死亡率) +主题:(病死率) + 主题:(流行) + 主题:(负担)+ 主题:(现况调查) + 主题:(现况研究))
时间：1990-2016
Search Terms: (subject: (nianlingxiangguanhuangbanbianxing) + subject: (nianlingxiangguanxinghuangbanbianxing) + subject: (laonianhuangbanbianxing) + subject: (laonianxinghuangbanbianxing) + subject: (nianlingxianghguanhuangbanbingbian)+ subject: (nianlingxiangguanxinghuangbanbingbian) + subject: (laonianhuangbanbingbian) + subject: (laonianxinghuangbanbingbian))* (subject: (fabinglv) + subject: (fashenglv) + subject: (huanbinglv)+ subject: (lihuanlv) + subject: (xianhuanlv) + subject: (siwanglv)+ subject: (bingsilv) + subject: (liuxing) + subject: (fudan)+ subject: (xiankuangdiaocha) + subject: (xiankuangyanjiu))
Date: 1990-2016

CBM-SinoMed
Access Date: 26 Jun 2016
Journal category: All journals
检索表达式：
((年龄相关黄斑变性) OR (年龄相关性黄斑变性) OR (老年黄斑变性) OR (老年性黄斑变性) OR (年龄相关黄斑病变) OR (年龄相关性黄斑病变) OR (老年黄斑病变) OR (老年性黄斑病变)) AND ((发病率) OR (发生率) OR (患病率) OR (罹患率) OR (现患率) OR (死亡率) OR (病死率) OR (流行) OR (负担) OR (现况调查) OR (现况研究))
时间：1990-2016
Search Terms: ((nianlingxiangguanhuangbanbianxing) OR (nianlingxiangguanxinghuangbanbianxing) OR (laonianhuangbanbianxing) OR (laonianxinghuangbanbianxing) OR (nianlingxianghguanhuangbanbingbian) OR (nianlingxiangguanxinghuangbanbingbian) OR (laonianhuangbanbingbian) OR (laonianxinghuangbanbingbian))* ((fabinglv) OR (fashenglv) OR (huanbinglv) OR (lihuanlv) OR (xianhuanlv) OR (siwanglv) OR (bingsilv) OR (liuxing) OR (fudan) OR (xiankuangdiaocha) OR (xiankuangyanjiu))
Date: 1990-2016

PubMed
Access Date: 16 Sep 2016
Search Terms:
((age-related macular degeneration OR age related macular degeneration OR age-related maculopathy OR age related maculopathy) AND (China OR Chinese OR Hongkong OR Macao OR Taiwan) AND (inciden* OR prevalen* OR morbidity OR mortality OR epidemiology)) AND ("1990/01/01"[Date - Publication] : "2016/09/17"[Date - Publication])

Embase (Ovid)
Access Date: 17 Sep 2016

#	Searches
1	age-related macular degeneration.mp. or exp retina macula age related degeneration/ or exp age related macular degeneration/ or exp retina macula degeneration/
2	exp retina maculopathy/ or age-related maculopathy.mp.
3	China.mp. or exp China/
4	exp Chinese/ or Chinese.mp.
5	Hong Kong.mp. or exp Hong Kong/
6	Macao.mp. or exp Macao/
7	Taiwan.mp. or exp Taiwan/
8	exp incidence/ or inciden*.mp.
9	exp prevalence/ or prevalen*.mp.
10	morbidity.mp. or morbidity/
11	mortality/ or Mortality.mp.
12	exp epidemiology/ or Epidemiology.mp.

13	1 or 2
14	3 or 4 or 5 or 6 or 7
15	8 or 9 or 10 or 11 or 12
16	13 and 14 and 15
17	limit 16 to yr="1990 -Current"

Medline (Ovid)
Access Date: 17 Sep 2016
Search Terms:

#	Searches
1	age-related macular degeneration.mp. or exp Macular Degeneration/
2	age-related maculopathy.mp.
3	China.mp. or exp China/
4	Chinese.mp.
5	Hong Kong.mp. or exp Hong Kong/
6	Macao.mp. or exp Macau/
7	Taiwan.mp. or exp Taiwan/
8	exp Incidence/ or inciden*.mp.
9	exp Prevalence/ or prevalen*.mp.
10	morbidity.mp. or exp Morbidity/
11	mortality.mp. or exp Mortality/
12	epidemiology.mp. or exp Epidemiology/
13	1 or 2
14	3 or 4 or 5 or 6 or 7
15	8 or 9 or 10 or 11 or 12
16	13 and 14 and 15
17	limit 16 to yr="1990 -Current"

Table S2. Meta-analysis of the prevalence of AMD for assessing heterogeneity between studies

To assess heterogeneity between studies that reported prevalence rates of AMD, standard meta-analyses were performed. First, the variances of the raw prevalence estimates were stabilized by using logit transformation. Then the heterogeneity between studies was assessed by the Cochran's Q and I^2 statistics, a p-value<0.05 indicates heterogeneity between studies in Q statistic, and I^2 represents the proportion of total variation that is due to heterogeneity rather than chance, where a value of 0% indicates no observed heterogeneity and values of 25%, 50% and 75% reflect low, moderate and high heterogeneity, respectively [60,61]. As shown in Figures S1-S4, significant high heterogeneity was indicated between studies that reported prevalence rates of any AMD (I^2=98.4%, p<0.0001), early AMD (I^2=98.7%, p<0.0001), late AMD (I^2=96.8%, p<0.0001), GA (I^2=90.5%, p<0.0001) and NVAMD (I^2=94.0%, p<0.0001).

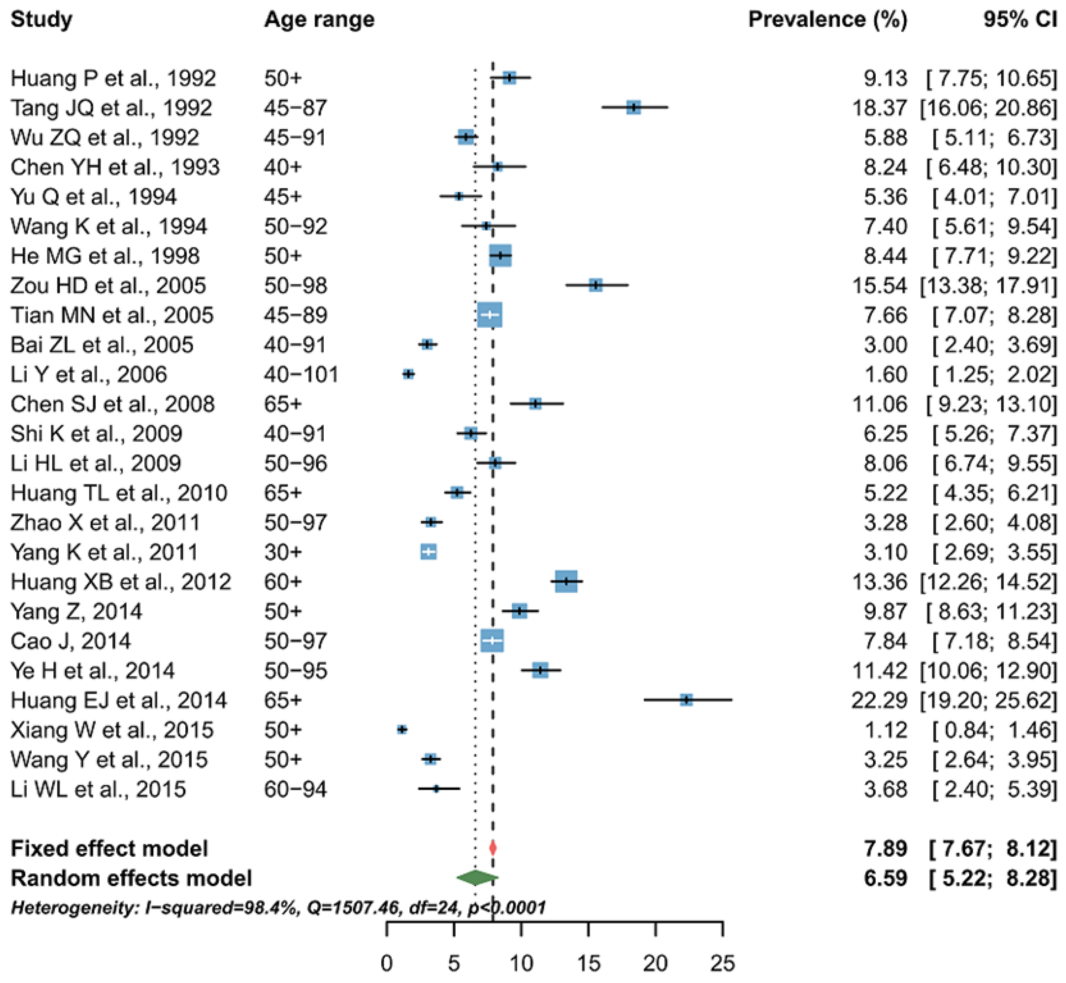

Figure S1. Meta-analysis of the prevalence of any AMD (n=25)

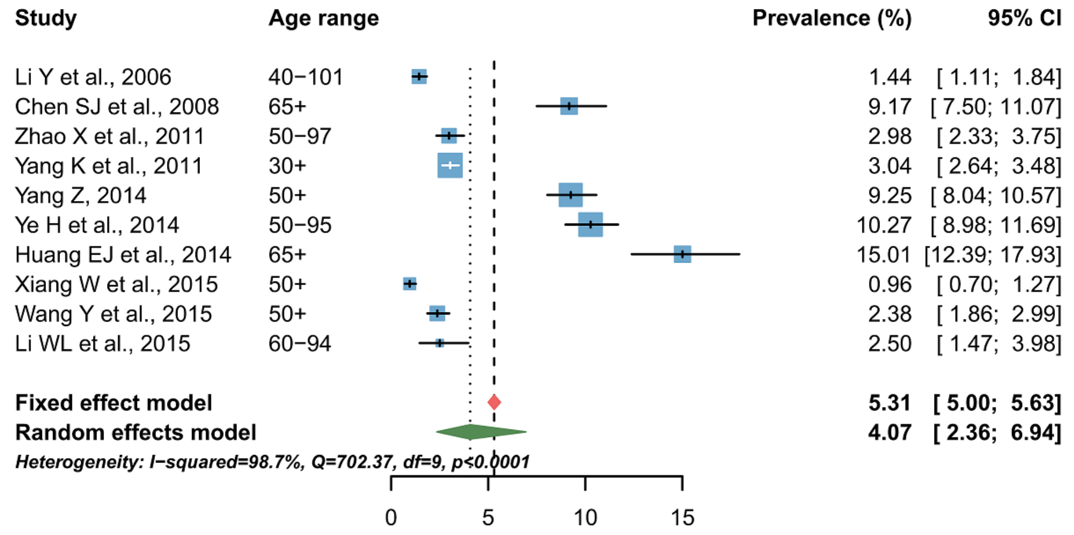

Figure S2. Meta-analysis of the prevalence of early AMD (n=9)

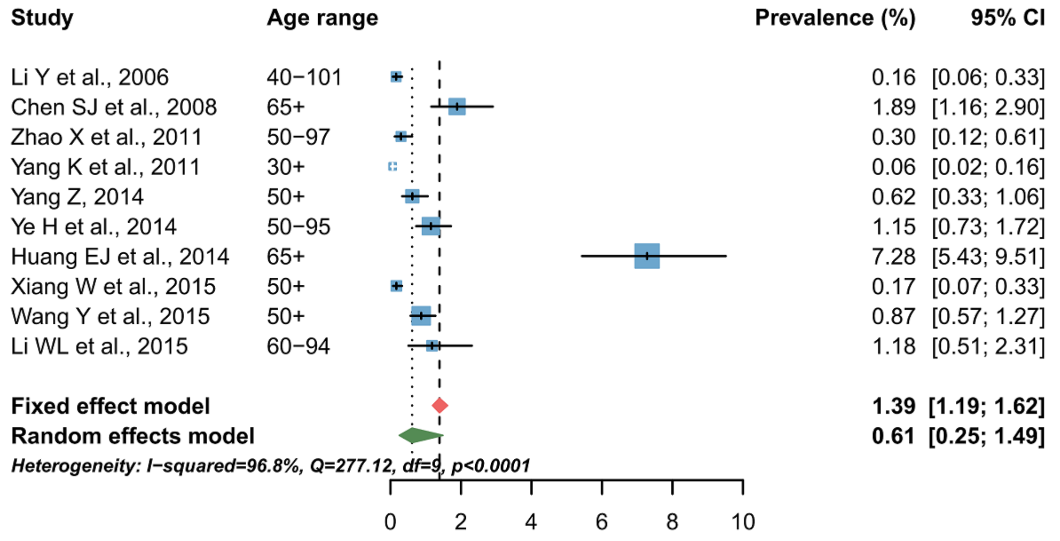

Figure S3. Meta-analysis of the prevalence of late AMD (n=9)

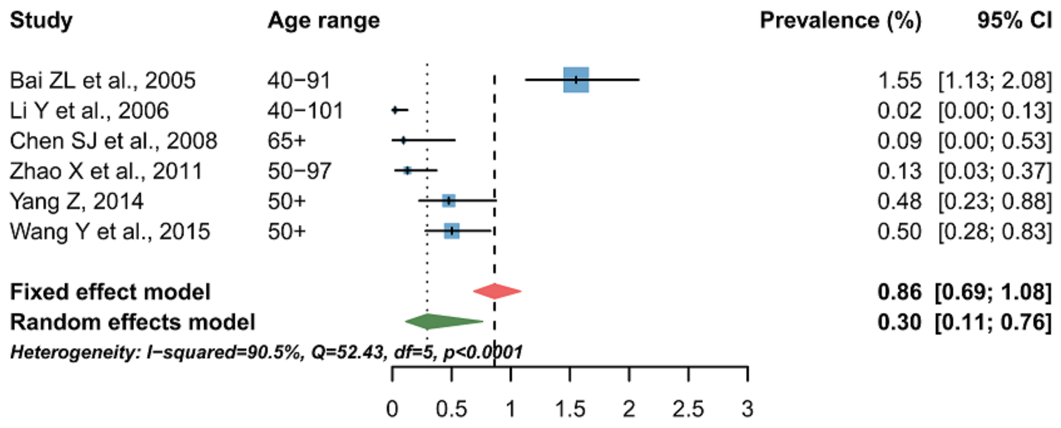

Figure S4. Meta-analysis of the prevalence of GA (n=6)

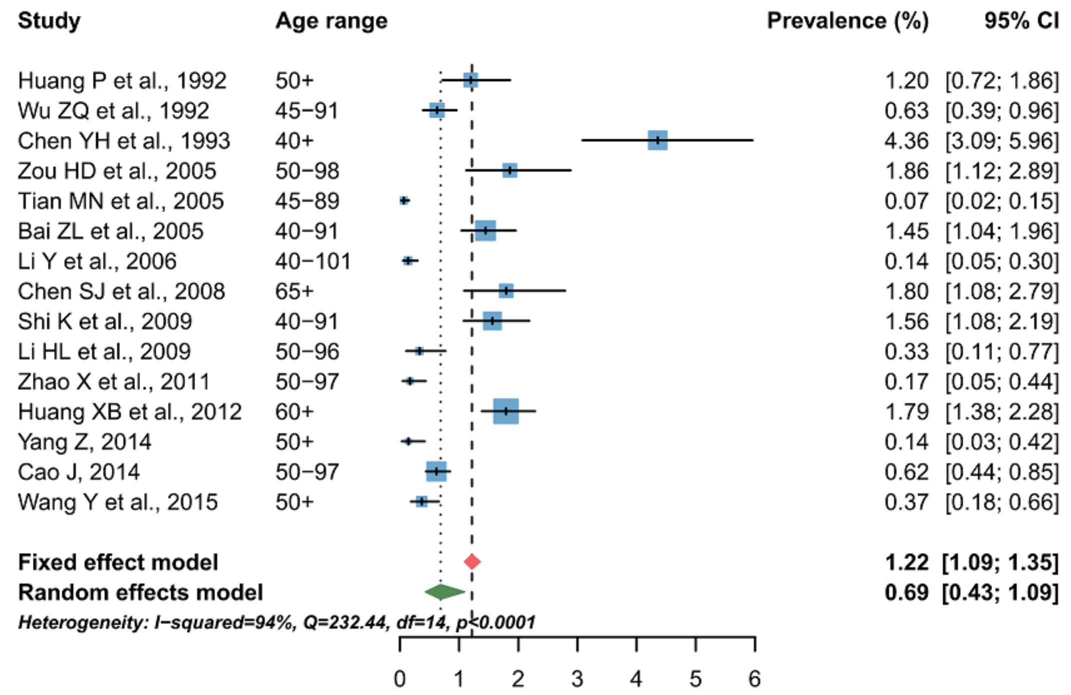

Figure S5. Meta-analysis of the prevalence of NVAMD (n=15)

Table S3. Full list of the included studies on AMD prevalence in China (n=25)

Study ID	Reference
**AMD-01**	Ping Huang, Ren-xiu He, Guo-zhen He, et al. 黄平，何仁秀，何国桢，等. The investigation of Age-related Macular Degeneration in Hunan Province* (湖南省老年黄斑变性流行病学调查) [J]. Chinese Ophthal Res (眼科研究). 1992(01): 60-61.
**AMD-02**	Jia-quan Tan, Ai-guang Nie, De-yong Jiang, et al. 谭家铨，聂爱光，姜德泳，等. The investigation of Age-related Macular Degeneration* (老年性黄斑变性流行病学调查) [J]. Hunan Medical Journal (湖南医学). 1992(05): 274-275.
**AMD-03**	Zheng-qing Wu, Jin-e Cao, Xiu-heng Yao, et al. 武正清，曹金娥，姚秀衡，等. Epidemiologic survey of senile macular degeneration (老年黄斑变性的流行病学调查) [J]. Chinese Journal of Ophthalmology (中华眼科杂志). 1992, 28(4): 246-247.
**AMD-04**	Yu-hua Chen, Ji-kui Shen, Yu-hong Zhang, et al. 陈玉华，申济奎，张宇弘，等. Senile macular degeneration in plateau aerea (高原地区老年性黄斑变性) [J]. Journal of High Altitude Medicine (实用眼科杂志). 1993(04): 57-58.
**AMD-05**	Qiang Yu, Jing-jing Xu, Si-ping Zhu, et al. 于强，许京京，朱斯平，等. Epidemiologic survey of Age-related Macular Degeneration in Doumen County, Guangdong province* (广东省斗门县老年黄斑变性流行病学调查) [J]. Chinese Journal of Ocular Fundus Diseases (中华眼底病杂志). 1994(2).
**AMD-06**	Kuang Wang, Jin Zhao, Jie-kai Jiang, et al. 王况，赵瑾，姜节凯，等. Epidemiologic survey of Age-related Macular Degeneration* (老年性黄斑变性的流行病学调查) [J]. Journal of Zhejiang Medical University (浙江医科大学学报). 1994(2).
**AMD-07**	Ming-guang He, Jing-jing Xu, Kai-li Wu, et al. 何明光，许京京，吴开力，等. The prevalence of age-related macular degeneration in Doumen county, Guangdong (广东省斗门县老年性黄斑变性流行病学调查) [J]. Chin J Ocul Fundus Dis (中华眼底病杂志). 1998(02): 61-63.
**AMD-08**	Hai-dong Zou, Zhe Zhang, Xun Xu, et al. 邹海东，张哲，许迅，等. Prevalence study of age-related macular degeneration in Caojiadu blocks, Shanghai (上海市静安区曹家渡街道年龄相关性黄斑变性的患病率调查) [J]. Chin J Ophthalmol (中华眼科杂志). 2005, 41(1): 15-19.
**AMD-09**	Man-nan Tian, Yue-mei Zhang, Li Li, et al. 田蔓男，张月梅，李丽，等. Epidemiologic survey of Age-related Macular Degeneration* (老年性黄斑变性的流行病学调查)[J]. Journal of Lanzhou University (Medical Sciences) (兰州大学学报（医学版）). 2005, 31(2): 70-71.
**AMD-10**	Zhi-lan Bai, Bai-chao Ren, Jian-gang Yang, et al. 白芝兰，任百超，杨建刚，等. Epidemiological investigation on age-related macular degeneration in rural area of Shaanxi Province, China (中国陕西省农村年龄相关性黄斑变性流行病学调查) [J]. International Journal of Ophthalmology (国际眼科杂志). 2005, 5(6): 1114-1121.
**AMD-11**	Kai Shi, Wen-fang Zhang, Xiao-yan Zhou, et al. 史凯，张文芳，周晓燕，等. Epidemiological investigation of ocular funds disease in Mongol above 40 years in Henan county (青海省河南县 40 岁以上世居蒙古族人群眼底病的流行病学调查) [J]. Chin Ophthal Res (眼科研究). 2009, 27(3): 239-242.
**AMD-12**	Hui-li Li, Ai-lin You, Di-ling Wan, et al. 李慧丽，犹爱林，万迪玲，等. Prevalence study of age-related macular degeneration in central urban area of Chongqing (重庆市主城区年龄相关性黄斑变性患病率调查) [J]. Chin J Pract Ophthalmol (中国实用眼科杂志). 2009, 27(12): 1425-1429.

**AMD-13**	Xin Zhao, Bi-qi Tian, Yun-he Hao, et al. 赵欣，田碧琪，郝云赫，等. Prevalence of age-related maculopathy in community of Xi Chang'an street of Beijing (北京西长安街社区 50 岁以上人群年龄相关性黄斑变性患病率调查) [J]. Int J Ophthalmol (国际眼科杂志). 2011, 11(8): 1364-1368.
**AMD-14**	Xiao-bo Huang, Hai-dong Zou, Ning Wang, et al. 黄晓波，邹海东，王宁，等. Prevalence of age-related macular degeneration in Beixinjing Community of Shanghai (上海市北新泾街道老年人年龄相关性黄斑变性的患病率调查) [J]. Journal of Shanghai Jiaotong University (Medical Science) (上海交通大学学报(医学版)). 2012(02): 155-159.
**AMD-15**	Zhen Yang 杨桢. Prevalence and Associated Risk Factors of Age-Related Macular Degeneration in 50 years and older Population in ShunQing District, NanChong (南充市顺庆区 50 岁及以上人群年龄相关性黄斑变性患病率及相关因素分析) [D]. North Sichuan Medical University (川北医学院), 2014.
**AMD-16**	Jia Cao 曹葭. Prevalence study of age-related macular degeneration over the age of 50's in Wuxi (无锡市 50 岁及以上人群年龄相关性黄斑变性流行病学调查)[D]. Nanjing Medical University (南京医科大学), 2014.
**AMD-17**	Wei Xiang, Hui-ping Li, Yang Liu, et al. 向伟，李慧平，刘洋，等. Prevalence Investigation of Age-related Macular Degeneration among Population Aged 50 Years or Above in Tongxin County of Ningxia (宁夏同心县≥50 岁人群年龄相关性黄斑变性患病率、危险因素及致盲情况分析) [J]. Journal of Ningxia Medical University (宁夏医科大学学报). 2015, 37(8): 927-930.
**AMD-18**	Ying Wang, Huai-jin Guan, Hong Lu, et al. 汪颖，管怀进，陆宏，等. The Prevalence of age-related macular degeneration in the rural area of Qidong County, Jiangsu Province (江苏省启东市农村地区老年性黄斑变性流行病学调查分析) [J]. Chin J Ocul Fundus Dis (中华眼底病杂志). 2015, 31(5): 459-461.
**AMD-19**	Wu-liang Li, Hui-ping Li, Na Li, et al. 李武靓，李慧平，李娜，等. Prevalence of age-related macular degeneration in elderly population in the rural area of Ningxia (宁夏农村地区 60 岁以上人群年龄相关性黄斑变性的患病率调查) [J]. Ningxia Medical Journal (宁夏医学杂志). 2015, 37(5): 401-404.
**AMD-20**	Li Y, Xu L, Jonas JB, et al. Prevalence of age-related maculopathy in the adult population in China: the Beijing eye study. AM J OPHTHALMOL 2006;142:788-793
**AMD-21**	Chen SJ, Cheng CY, Peng KL, et al. Prevalence and associated risk factors of age-related macular degeneration in an elderly Chinese population in Taiwan: the Shihpai Eye Study. Invest Ophthalmol Vis Sci 2008;49:3126-3133
**AMD-22**	Huang TL, Hsu SY, Tsai RK, Sheu MM. Etiology of ocular diseases in elderly Amis aborigines in Eastern Taiwan (The Amis Eye Study). JPN J OPHTHALMOL 2010;54:266-271
**AMD-23**	Yang K, Liang YB, Gao LQ, et al. Prevalence of age-related macular degeneration in a rural Chinese population: the Handan Eye Study. OPHTHALMOLOGY 2011;118:1395-1401
**AMD-24**	Ye H, Zhang Q, Liu X, et al. Prevalence of age-related macular degeneration in an elderly urban chinese population in China: the Jiangning Eye Study. Invest Ophthalmol Vis Sci 2014;55:6374-6380
**AMD-25**	Huang EJ, Wu SH, Lai CH, et al. Prevalence and risk factors for age-related macular degeneration in the elderly Chinese population in south-western Taiwan: the Puzih eye study. Eye (Lond) 2014;28:705-714

Note: The Chinese publication list employed the journals' official English names or abbreviations, English titles were obtained from journals or literature databases (CNKI, Wanfang and CBM). Where official English translation of journal names is not available, a pinyin title is adopted; where the English translation of titles is not available, I translated the titles, labelled with "" and marked as green.*

Table S4. Detailed characteristics of the included studies on AMD prevalence in China (n=25)

Study ID	Study	Province	Setting	Gender	Study year	Asses sment	Grading system	Sample size	Any AMD	Early AMD	Late AMD	GA	NVA MD
**AMD-01**	Huang P et al. (1992)	Hunan	Mixed	Both	1987	FI	CMA1986	1589	145	-	-	-	19
**AMD-02**	Tang JQ et al. (1992)	Hunan	Mixed	Both	1989	FI	CMA1986	1040	191	-	-	-	-
**AMD-03**	Wu ZQ et al. (1992)	Hunan	Mixed	Both	1989	FI	CMA1986	3351	197	-	-	-	21
**AMD-04**	Chen YH et al. (1993)	Shaanxi Qinghai	Mixed	Mixed	1991	FI	CMA1986	849	70	-	-	-	37
**AMD-05**	Yu Q et al. (1994)	Guangdong	Rural	Both	1990	FI	CMA1986	932	50	-	-	-	-
**AMD-06**	Wang K et al. (1994)	Zhejiang	Mixed	Both	1989	FI	CMA1986	730	54	-	-	-	-
**AMD-07**	He MG et al. (1998)	Guangdong	Rural	Both	1997	FI	CMA1986	5342	451	-	-	-	-
**AMD-08**	Zou HD et al. (2005)	Shanghai	Urban	Both	2003	FI	CMA1986	1023	159	-	-	-	19
**AMD-09**	Tian MN et al. (2005)	Gansu	Mixed	Mixed	2002	FI	CMA1986	7563	579	-	-	-	5
**AMD-10**	Bai ZL et al. (2005)	Shaanxi	Rural	Both	2003	FI	IC	2835	85	-	-	44	41
**AMD-11**	Shi K et al. (2009)	Qinghai	Rural	Mixed	2006	FI	CMA1986	2112	132	-	-	-	33
**AMD-12**	Li HL et al. (2009)	Chongqing	Urban	Both	2009	FI	"Ophthalmology" (7th version)	1513	122	-	-	-	5
**AMD-13**	Zhao X et al. (2011)	Beijing	Urban	Both	2006	FI	CARMS	2348	77	70	7	3	4
**AMD-14**	Huang XB et al. (2012)	Shanghai	Urban	Both	2008	FI	CMA1986	3571	477	-	-	-	64
**AMD-15**	Yang Z (2014)	Sichuan	Urban	Both	2013	FI	CARMS	2097	207	194	13	10	3
**AMD-16**	Cao J (2014)	Jiangsu	Urban	Both	2010	FI	CMA1986	6150	482	-	-	-	38
**AMD-17**	Xiang W et al. (2015)	Ningxia	Rural	Both	2014	FI	CARMS	4812	54	46	8	-	-
**AMD-18**	Wang Y et al. (2015)	Jiangsu	Rural	Both	2015	FI	CARMS	2985	97	71	26	15	11
**AMD-19**	Li WL et al. (2015)	Ningxia	Rural	Both	2013	FI	CARMS	679	25	17	8	-	-
**AMD-20**	Li Y et al. (2006)	Beijing	Mixed	Mixed	2001	FI	WARM	4376	70	63	7	1	6
**AMD-21**	Chen SJ et al. (2008)	Taiwan	Mixed	Both	1999	FI	WARM	1058	117	97	20	1	19
**AMD-22**	Huang TL et al. (2010)	Taiwan	Rural	Both	2007	FI	WARM	2316	121	-	-	-	-
**AMD-23**	Yang K et al. (2011)	Hebei	Rural	Mixed	2006	FI	WARM	6581	204	200	4	-	-
**AMD-24**	Ye H et al. (2014)	Shanghai	Urban	Both	2013	FI	WARM	2005	229	206	23	-	-

**AMD-25**	Huang EJ et al. (2014)	Taiwan	Mixed	Both	2011	FI	WARM	673	150	101	49	-	-

Note: "-" represents unavailable data; FI, Fundus imaging; CAM 1986, the "Age-related Macular Degeneration Clinical Diagnosis Standard" proposed by the China Medical Association in 1986; WARMGS, the Wisconsin age-related maculopathy system; CARMS, the Clinical Age-Related Maculopathy Grading System; IC, the International Classification and Grading system

Table S5. Estimate and projection of the national age-specific number of people with any AMD, early AMD, late AMD, GA and NVAMD in China from 1990 to 2050 (million, 95% CI)

Age	Any AMD	Early AMD	Late AMD	GA	NVAMD
**1990**					
**Age**	**Any AMD**	**Early AMD**	**Late AMD**	**GA**	**NVAMD**
45-49 years	1.19 (0.90-1.56)	1.09 (0.87-1.32)	0.23 (0.13-0.42)	0.09 (0.04-0.20)	0.14 (0.09-0.22)
50-54 years	1.45 (1.11-1.90)	1.27 (1.02-1.52)	0.29 (0.17-0.51)	0.11 (0.05-0.23)	0.18 (0.12-0.27)
55-59 years	1.71 (1.32-2.23)	1.42 (1.16-1.70)	0.35 (0.21-0.59)	0.13 (0.06-0.26)	0.22 (0.15-0.33)
60-64 years	1.78 (1.37-2.29)	1.41 (1.16-1.67)	0.37 (0.23-0.61)	0.13 (0.06-0.26)	0.24 (0.17-0.35)
65-69 years	1.75 (1.35-2.24)	1.33 (1.09-1.56)	0.37 (0.23-0.61)	0.12 (0.06-0.25)	0.25 (0.17-0.35)
70-74 years	1.73 (1.35-2.21)	1.26 (1.05-1.47)	0.38 (0.24-0.62)	0.12 (0.06-0.25)	0.26 (0.18-0.37)
75-79 years	1.32 (1.03-1.67)	0.93 (0.77-1.07)	0.30 (0.19-0.50)	0.09 (0.04-0.19)	0.21 (0.14-0.30)
80–84 years	0.77 (0.60-0.96)	0.52 (0.44-0.60)	0.19 (0.11-0.31)	0.05 (0.02-0.12)	0.13 (0.09-0.19)
85-89 years	0.32 (0.25-0.40)	0.21 (0.18-0.24)	0.08 (0.05-0.14)	0.02 (0.01-0.05)	0.06 (0.04-0.09)
Total (45-89 years)	12.01 (9.29-15.46)	9.44 (7.74-11.15)	2.58 (1.56-4.30)	0.87 (0.40-1.83)	1.71 (1.16-2.47)
**2000**					
**Age**	**Any AMD**	**Early AMD**	**Late AMD**	**GA**	**NVAMD**
45-49 years	2.09 (1.59-2.75)	1.91 (1.53-2.32)	0.41 (0.23-0.75)	0.16 (0.07-0.36)	0.25 (0.16-0.39)
50-54 years	1.94 (1.48-2.54)	1.69 (1.37-2.03)	0.39 (0.23-0.67)	0.14 (0.07-0.31)	0.24 (0.16-0.36)
55-59 years	1.91 (1.47-2.48)	1.59 (1.29-1.89)	0.39 (0.23-0.66)	0.14 (0.06-0.29)	0.25 (0.17-0.36)
60-64 years	2.23 (1.72-2.88)	1.77 (1.45-2.09)	0.47 (0.29-0.77)	0.16 (0.08-0.33)	0.31 (0.21-0.43)
65-69 years	2.42 (1.88-3.11)	1.84 (1.52-2.16)	0.52 (0.32-0.84)	0.17 (0.08-0.35)	0.35 (0.24-0.49)
70-74 years	2.19 (1.71-2.79)	1.59 (1.32-1.85)	0.48 (0.30-0.78)	0.15 (0.07-0.32)	0.33 (0.23-0.47)
75-79 years	1.72 (1.35-2.18)	1.21 (1.00-1.40)	0.40 (0.24-0.65)	0.12 (0.06-0.25)	0.28 (0.19-0.39)
80–84 years	1.23 (0.97-1.55)	0.84 (0.70-0.96)	0.30 (0.18-0.49)	0.09 (0.04-0.19)	0.21 (0.14-0.31)
85-89 years	0.58 (0.46-0.72)	0.38 (0.32-0.44)	0.15 (0.09-0.25)	0.04 (0.02-0.09)	0.11 (0.07-0.16)
Total (45-89 years)	16.31 (12.62-20.99)	12.81 (10.51-15.13)	3.50 (2.11-5.86)	1.18 (0.54-2.50)	2.32 (1.57-3.36)
**2010**					
**Age**	**Any AMD**	**Early AMD**	**Late AMD**	**GA**	**NVAMD**
45-49 years	2.47 (1.87-3.25)	2.26 (1.81-2.75)	0.49 (0.27-0.88)	0.19 (0.08-0.42)	0.30 (0.19-0.46)
50-54 years	2.60 (1.98-3.40)	2.27 (1.83-2.72)	0.52 (0.03-0.90)	0.19 (0.09-0.42)	0.33 (0.21-0.49)

55-59 years	3.42 (2.63-4.44)	2.84 (2.32-3.39)	0.70 (0.42-1.18)	0.25 (0.12-0.53)	0.45 (0.30-0.65)
60-64 years	3.06 (2.36-3.95)	2.43 (1.99-2.87)	0.64 (0.39-1.05)	0.22 (0.10-0.45)	0.42 (0.29-0.60)
65-69 years	2.81 (2.18-3.60)	2.13 (1.76-2.50)	0.60 (0.37-0.98)	0.20 (0.09-0.41)	0.40 (0.28-0.57)
70-74 years	2.90 (2.26-3.70)	2.11 (1.75-2.46)	0.64 (0.40-1.04)	0.20 (0.10-0.42)	0.44 (0.30-0.62)
75-79 years	2.58 (2.02-3.26)	1.81 (1.51-2.09)	0.59 (0.37-0.97)	0.18 (0.08-0.38)	0.41 (0.28-0.59)
80–84 years	1.72 (1.36-2.16)	1.17 (0.98-1.35)	0.42 (0.25-0.69)	0.12 (0.05-0.26)	0.30 (0.20-0.43)
85-89 years	0.88 (0.70-1.09)	0.58 (0.49-0.66)	0.22 (0.13-0.38)	0.06 (0.03-0.14)	0.16 (0.11-0.24)
Total (45-89 years)	22.43 (17.36-28.85)	17.60 (14.45-20.78)	4.83 (2.91-8.07)	1.62 (0.75-3.43)	3.21 (2.16-4.64)
			**2015**		
**Age**	**Any AMD**	**Early AMD**	**Late AMD**	**GA**	**NVAMD**
45-49 years	3.02 (2.29-3.97)	2.76 (2.22-3.36)	0.59 (0.33-1.08)	0.23 (0.10-0.52)	0.36 (0.23-0.56)
50-54 years	3.19 (2.43-4.16)	2.78 (2.25-3.34)	0.64 (0.37-1.11)	0.24 (0.11-0.51)	0.40 (0.26-0.59)
55-59 years	3.31 (2.54-4.30)	2.75 (2.25-3.28)	0.68 (0.41-1.14)	0.24 (0.11-0.51)	0.43 (0.29-0.63)
60-64 years	4.26 (3.29-5.50)	3.38 (2.78-3.99)	0.89 (0.55-1.46)	0.31 (0.14-0.63)	0.58 (0.40-0.83)
65-69 years	3.64 (2.82-4.67)	2.76 (2.28-3.24)	0.78 (0.48-1.27)	0.26 (0.12-0.53)	0.52 (0.36-0.74)
70-74 years	3.10 (2.42-3.96)	2.26 (1.88-2.63)	0.69 (0.43-1.11)	0.22 (0.10-0.45)	0.47 (0.32-0.66)
75-79 years	2.85 (2.24-3.61)	2.00 (1.67-2.32)	0.66 (0.41-1.07)	0.20 (0.09-0.42)	0.46 (0.31-0.65)
80–84 years	2.15 (1.69-2.70)	1.46 (1.22-1.68)	0.52 (0.31-0.86)	0.15 (0.07-0.33)	0.37 (0.25-0.53)
85-89 years	1.13 (0.89-1.40)	0.75 (0.63-0.85)	0.29 (0.17-0.49)	0.08 (0.04-0.18)	0.21 (0.14-0.31)
Total (45-89 years)	26.65 (20.62-34.27)	20.91 (17.16-24.68)	5.74 (3.46-9.59)	1.93 (0.89-4.08)	3.81 (2.57-5.51)
			**2020**		
**Age**	**Any AMD**	**Early AMD**	**Late AMD**	**GA**	**NVAMD**
45-49 years	2.88 (2.19-3.79)	2.65 (2.12-3.22)	0.57 (0.32-1.03)	0.22 (0.10-0.50)	0.35 (0.22-0.54)
50-54 years	3.90 (2.98-5.10)	3.41 (2.76-4.10)	0.78 (0.46-1.36)	0.29 (0.13-0.63)	0.49 (0.32-0.73)
55-59 years	4.07 (3.13-5.28)	3.39 (2.77-4.04)	0.83 (0.50-1.40)	0.30 (0.14-0.63)	0.54 (0.36-0.77)
60-64 years	4.14 (3.20-5.34)	3.29 (2.71-3.89)	0.87 (0.53-1.43)	0.30 (0.14-0.62)	0.57 (0.39-0.81)
65-69 years	5.11 (3.97-6.55)	3.89 (3.22-4.56)	1.10 (0.68-1.79)	0.36 (0.17-0.75)	0.74 (0.51-1.04)
70-74 years	4.08 (3.18-5.21)	2.99 (2.48-3.48)	0.91 (0.56-1.47)	0.29 (0.14-0.59)	0.62 (0.43-0.88)
75-79 years	3.12 (2.45-3.96)	2.20 (1.83-2.55)	0.72 (0.45-1.18)	0.22 (0.10-0.46)	0.50 (0.34-0.72)

80–84 years	2.45 (1.93-3.08)	1.67 (1.40-1.92)	0.60 (0.36-0.98)	0.17 (0.08-0.37)	0.42 (0.28-0.61)
85-89 years	1.46 (1.16-1.83)	0.97 (0.82-1.11)	0.38 (0.22-0.64)	0.11 (0.05-0.23)	0.27 (0.18-0.40)
Total (45-89 years)	31.23 (24.18-40.14)	24.47 (20.1-28.87)	6.76 (4.08-11.28)	2.26 (1.04-4.78)	4.50 (3.04-6.50)
**2030**					
**Age**	**Any AMD**	**Early AMD**	**Late AMD**	**GA**	**NVAMD**
45-49 years	2.38 (1.80-3.13)	2.21 (1.78-2.69)	0.48 (0.27-0.86)	0.18 (0.08-0.41)	0.29 (0.19-0.45)
50-54 years	2.97 (2.26-3.88)	2.63 (2.13-3.16)	0.60 (0.35-1.05)	0.22 (0.10-0.49)	0.38 (0.25-0.56)
55-59 years	4.79 (3.68-6.21)	4.04 (3.30-4.81)	0.99 (0.60-1.67)	0.36 (0.17-0.75)	0.64 (0.43-0.92)
60-64 years	6.29 (4.86-8.12)	5.08 (4.17-5.99)	1.34 (0.82-2.20)	0.46 (0.22-0.95)	0.88 (0.60-1.25)
65-69 years	6.22 (4.83-7.98)	4.80 (3.97-5.62)	1.36 (0.84-2.20)	0.45 (0.21-0.92)	0.91 (0.63-1.28)
70-74 years	5.76 (4.49-7.35)	4.27 (3.54-4.97)	1.30 (0.81-2.10)	0.41 (0.19-0.85)	0.89 (0.61-1.25)
75-79 years	6.10 (4.78-7.73)	4.36 (3.63-5.04)	1.43 (0.88-2.33)	0.44 (0.20-0.91)	1.00 (0.68-1.42)
80–84 years	3.84 (3.03-4.83)	2.66 (2.22-3.05)	0.95 (0.57-1.56)	0.28 (0.12-0.59)	0.67 (0.45-0.97)
85-89 years	2.05 (1.62-2.55)	1.38 (1.15-1.57)	0.53 (0.31-0.90)	0.15 (0.06-0.33)	0.38 (0.25-0.57)
Total (45-89 years)	40.40 (31.36-51.78)	31.42 (25.90-36.90)	8.98 (5.45-14.87)	2.95 (1.36-6.20)	6.03 (4.09-8.67)
**2040**					
**Age**	**Any AMD**	**Early AMD**	**Late AMD**	**GA**	**NVAMD**
45-49 years	2.52 (1.91-3.32)	2.37 (1.91-2.87)	0.51 (0.29-0.92)	0.20 (0.09-0.44)	0.31 (0.20-0.48)
50-54 years	4.00 (3.05-5.23)	3.58 (2.91-4.29)	0.82 (0.48-1.42)	0.31 (0.14-0.66)	0.52 (0.34-0.76)
55-59 years	3.97 (3.05-5.16)	3.39 (2.77-4.02)	0.83 (0.50-1.40)	0.30 (0.14-0.62)	0.53 (0.36-0.77)
60-64 years	4.83 (3.73-6.24)	3.93 (3.24-4.63)	1.04 (0.64-1.70)	0.36 (0.17-0.73)	0.68 (0.47-0.96)
65-69 years	7.46 (5.79-9.56)	5.81 (4.81-6.79)	1.64 (1.02-2.66)	0.54 (0.26-1.11)	1.10 (0.76-1.55)
70-74 years	9.08 (7.08-11.57)	6.79 (5.65-7.88)	2.07 (1.29-3.33)	0.66 (0.31-1.35)	1.41 (0.98-1.99)
75-79 years	7.88 (6.17-9.98)	5.68 (4.74-6.55)	1.87 (1.15-3.03)	0.57 (0.26-1.18)	1.30 (0.89-1.85)
80–84 years	5.93 (4.67-7.46)	4.14 (3.47-4.74)	1.47 (0.89-2.43)	0.43 (0.19-0.92)	1.04 (0.70-1.51)
85-89 years	4.55 (3.61-5.67)	3.10 (2.60-3.52)	1.20 (0.71-2.01)	0.34 (0.15-0.74)	0.86 (0.56-1.27)
Total (45-89 years)	50.22 (39.07-64.18)	38.78 (32.10-45.28)	11.45 (6.97-18.90)	3.69 (1.71-7.76)	7.76 (5.26-11.13)
**2050**					
**Age**	**Any AMD**	**Early AMD**	**Late AMD**	**GA**	**NVAMD**
45-49 years	1.77 (1.35-2.33)	1.68 (1.36-2.03)	0.36 (0.20-0.65)	0.14 (0.06-0.31)	0.22 (0.14-0.34)

50-54 years	2.42 (1.85-3.17)	2.19 (1.78-2.62)	0.50 (0.29-0.87)	0.19 (0.08-0.40)	0.32 (0.21-0.47)
55-59 years	4.23 (3.25-5.50)	3.65 (2.99-4.32)	0.90 (0.54-1.50)	0.32 (0.15-0.67)	0.58 (0.39-0.83)
60-64 years	6.58 (5.08-8.49)	5.41 (4.47-6.35)	1.42 (0.88-2.33)	0.49 (0.23-1.01)	0.93 (0.65-1.32)
65-69 years	6.29 (4.88-8.07)	4.95 (4.12-5.77)	1.40 (0.87-2.26)	0.46 (0.22-0.94)	0.94 (0.65-1.32)
70-74 years	7.19 (5.60-9.16)	5.43 (4.53-6.29)	1.65 (1.03-2.66)	0.52 (0.25-1.07)	1.13 (0.78-1.58)
75-79 years	9.95 (7.80-12.60)	7.25 (6.07-8.34)	2.38 (1.48-3.86)	0.72 (0.34-1.51)	1.66 (1.14-2.35)
80–84 years	10.15 (7.99-12.76)	7.16 (6.01-8.18)	2.55 (1.55-4.18)	0.74 (0.34-1.59)	1.80 (1.21-2.60)
85-89 years	6.60 (5.23-8.23)	4.54 (3.82-5.15)	1.75 (1.04-2.94)	0.49 (0.22-1.08)	1.26 (0.82-1.86)
Total (45-89 years)	55.19 (43.04-70.30)	42.26 (35.15-49.05)	12.92 (7.89-21.26)	4.09 (1.89-8.59)	8.84 (6.00-12.66)

Table S6. Meta-regression of AMD prevalence (logit form), univariable analyses

Variable	Number of studies	β (95 % CI)	p value
**Any AMD**			
Intercept	25	[-2.489] ([-2.764]-[-2.215])	<0.001
Age	25	0.056 (0.052- 0.060)	<0.001
Gender-male$	19	0.069 ([-0.004]-0.141)	0.063
Setting-rural	16	[-0.829] ([-1.304]-[-0.354])	<0.001
Latitude	25	[-0.076] ([-0.119]-[-0.033])	<0.001
Longitude	25	0.023 ([-0.017]-0.064)	0.262
Insolation	25	[-0.551] ([-1.132]-0.030)	0.063
Investigation year	25	[-0.014] ([-0.045]-0.017)	0.370
**Early AMD**			
Intercept	10	[-2.982] ([-3.528]-[-2.436])	<0.001
Age	10	0.045 (0.039-0.052)	<0.001
Gender-male$	7	0.197 (0.054-0.339)	0.007
Setting-rural	7	[-1.037] ([-1.833]-[-0.241])	0.011
Latitude	10	[-0.127] ([-0.181]-[-0.072])	<0.001
Longitude	10	0.052 ([-0.033]-0.137)	0.233
Insolation	10	[-0.864] ([-2.155]-0.428)	0.190
Investigation year	10	0.003 ([-0.103]-0.109)	0.954
**Late AMD**			
Intercept	10	[-4.548] ([-5.308]-[-3.787])	<0.001
Age	10	0.071 (0.055-0.088)	<0.001
Gender-male$	7	0.321 ([-0.053]-0.696)	0.093
Setting-rural	7	[-0.229] ([-1.715]-1.257)	0.763
Latitude	10	[-0.167] ([-0.247]-[-0.086])	<0.001
Longitude	10	0.045 ([-0.078]-0.169)	0.472
Insolation	10	0.066 ([-1.915]-2.046)	0.948
Investigation year	10	0.054 ([-0.090]-0.198)	0.464
**GA**			
Intercept	6	[-5.517] ([-6.557]-[-4.478])	<0.001
Age	6	0.050 (0.028-0.072)	<0.001
Gender-male$	2	0.089 ([-0.449]-0.627)	0.746
Setting-rural	4	1.261 ([-0.734]-3.255)	0.215
Latitude	6	[-0.048] ([-0.274]-0.179)	0.681
Longitude	6	[-0.115] ([-0.280]-0.050)	0.173
Insolation	6	[-1.522] ([-3.914]-0.871)	0.213
Investigation year	6	0.081 ([-0.105]-0.266)	0.394
**NVAMD**			
Intercept	15	[-4.756] ([-5.360]-[-4.152])	<0.001
Age	15	0.063 (0.046-0.079)	<0.001
Gender-male$	6	0.376 (0.070-0.682)	0.016
Setting-rural	9	0.433 ([-0.827]-1.693)	0.501
Latitude	15	0.074 ([-0.061]-0.209)	0.282
Longitude	15	[-0.078] ([-0.142]-[-0.014])	0.017
Insolation	15	0.973 (0.290-1.656)	0.005
Investigation year	15	[-0.057] ([-0.133]-0.020)	0.149

Note: $ the estimate of gender effect was based on studies that provided AMD prevalence for both males and females; coefficients represent log odds ratios (ORs).

REFERENCES - SUPPLEMENTARY ONLINE MATERIAL

60. Higgins J, Thompson SG. Quantifying heterogeneity in a meta-analysis. Stat Med. 2002;21:1539-1558.
61. Higgins JP, Thompson SG, Deeks JJ, Altman DG. Measuring inconsistency in meta-analyses. Bmj. 2003;327:557-560.
